# Machine learning–based skin nerve morphometry for diabetic neuropathy: diagnostic and clinical implications

**DOI:** 10.1093/braincomms/fcag113

**Published:** 2026-03-31

**Authors:** Hsueh-Wen Hsueh, Yao-Yu Wu, Tzu-I Chuang, Cheng-Chen Lin, Ti-Yen Yeh, Yi-Hui Kao, Herng-Hua Chang, Chi-Chao Chao, Sung-Tsang Hsieh

**Affiliations:** Department of Neurology, National Taiwan University Hospital, Taipei 100225, Taiwan; Department of Anatomy and Cell Biology, National Taiwan University College of Medicine, Taipei 100225, Taiwan; Department of Neurology, National Taiwan University Hospital Hsin-Chu Branch, Hsin-Chu 302058, Taiwan; School of Medicine, National Taiwan University, Taipei 100225, Taiwan; School of Medicine, National Taiwan University, Taipei 100225, Taiwan; Department of Anatomy and Cell Biology, National Taiwan University College of Medicine, Taipei 100225, Taiwan; Department of Anatomy and Cell Biology, National Taiwan University College of Medicine, Taipei 100225, Taiwan; Department of Anatomy and Cell Biology, National Taiwan University College of Medicine, Taipei 100225, Taiwan; Department of Neurology, National Taiwan University Hospital Yunlin Branch, Yunlin 640, Taiwan; Department of Medical Education and Research, National Taiwan University Hospital Yun-Lin Branch, Douliu 640, Taiwan; Department of Engineering Science and Ocean Engineering, National Taiwan University, Taipei 106319, Taiwan; Department of Neurology, National Taiwan University Hospital, Taipei 100225, Taiwan; Department of Neurology, National Taiwan University Hospital, Taipei 100225, Taiwan; Department of Anatomy and Cell Biology, National Taiwan University College of Medicine, Taipei 100225, Taiwan

**Keywords:** diabetic polyneuropathy, machine learning, small fibre assessment, neuropathy, cutaneous nerve

## Abstract

This study aimed to (i) develop and validate new intraepidermal nerve fibres (IENFs) biomarkers with the aid of machine learning algorithms for the diagnosis of small-fibre neuropathy in diabetic patients and (ii) explore the diagnostic performance and clinical significance of these new biomarkers. Patients with diabetic neuropathy and control subjects were recruited. Area-based morphometry of IENF (IENFa) parameters were developed by using the machine learning system for automatic quantification. The diagnostic performance was assessed according to receiver operating characteristic analysis. The clinical implications of the various IENFa parameters were examined by exploring their correlations with metabolic profiles and via electrophysiological experiments. The diabetic neuropathy (*n* = 48) and control (*n* = 63) cohorts were comparable in terms of age and sex. The IENFa parameters were inversely correlated with age, and only the IENF density (IENFd, the number of fibres per unit length of epidermis) and IENFa/A parameters were observed to be sex dependent in the control group. All of the IENFa parameters demonstrated equivalent performance according to (i) the correlation with IENFd and (ii) the diagnosis of IENFd-based small-fibre neuropathy by the receiver operating characteristic analysis (area under curve: 0.91–0.95, *P* > 0.05). Furthermore, the IENFa biomarkers were significantly correlated with sural sensory nerve action potential amplitudes. In summary, automatic IENFa is time-efficient and performs comparably to IENFd in diagnosing diabetic small-fibre neuropathy with high reliability. Furthermore, the IENFa parameter reflects concurrent large-fibre involvement in diabetic neuropathy. As the IENFa represents the total area of all IENFs, the results also imply global axonal atrophy in diabetic neuropathy.

## Teaser

This study developed a machine learning–based quantification platform for intraepidermal nerve fibres on skin biopsies to diagnose small-fibre neuropathy in diabetes. The new measure showed strong correlations with conventional parameter of skin innervation and neurophysiological examinations, demonstrating comparable performance and clinical relevance for assessing nerve involvement in diabetic neuropathy.

## Graphical Abstract

**Graphical Abstract:**
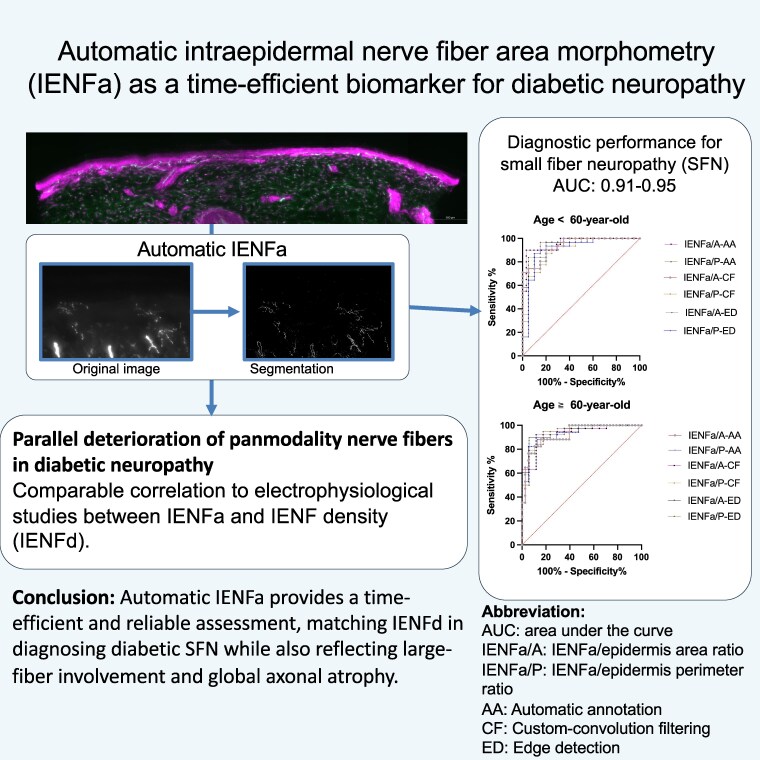

## Introduction

Diabetic neuropathy is a disabling complication leading to a significant disease burden among affected individuals and mainly affects small-diameter nociceptive and autonomic nerve fibres during the early phase.^1-5^ In the advanced stage, large-diameter nerve fibres are also involved.^1-3^ Small-fibre neuropathy is characterized by positive manifestations (such as neuropathic pain and touch-induced allodynia) and negative symptoms (including impairments in thermal sensation and painful stimuli).^1-4^ As the nerve terminals of nociceptive fibres reside in the epidermis of the skin, the documentation of nerve degeneration in patients with impaired thermal and nociceptive functions requires skin biopsy to quantify intraepidermal nerve fibres (IENFs).^6-8^ The reduction in IENF density (IENFd) reflects the consequence of nerve degeneration according to ultrastructural studies, thus providing pathological evidence of small-fibre neuropathy.^6-9^

Currently, skin biopsy combined with the quantification of IENFd represents the classical standard for the diagnosis of small-fibre neuropathy.^6-11^ Such an approach relies on manually counting the number of IENFs according to the consensus and guidelines.^6-9,11-13^ Despite the wide applications of IENFd, there is room for improvement regarding the assessment of small-fibre neuropathy, including whether there are additional attributes of IENFs in addition to the number to reflect skin innervation.^13^ One particular scenario is whether the total area of the IENFs is similar in subjects with the same IENFd values.

With advances in AI-based technologies, the interpretation and analysis of histopathological slides have transitioned into a new era.^14-17^ The applications of machine learning and computer vision algorithms have revolutionized the quantitative analysis of images and generated new insights that were not previously recognized via manual operation and visual inspections of the data and images.^14,15,18,19^ These technological improvements have resulted in a promising approach via the digitization of the images of skin biopsies and the application of algorithms involving machine learning–based automatic direct segmentation of skin innervation to (i) design new area-based parameters of IENFs and (ii) explore the clinical significance and implications.

Thus, this study aimed to develop IENF area (IENFa)–based parameters (designated as IENFa) by applying machine learning algorithms to automatically quantify skin innervation for diabetic neuropathy. Specifically, we addressed the following important issues for area-based parameters: (i) whether the diagnostic performance of IENFa is similar to that of IENFd and (ii) whether IENFa could serve as a new biomarker exhibiting clinical significance.

## Materials and methods

### Subject recruitment

This study was approved by the Institutional Review Boards (IRBs) of the National Taiwan University Hospital (200906026R, 201705058RIND and 201801087RINC) and registered at ClinicalTrials.gov (NCT05048862, registered date: 2021-08-01). The subjects included (i) a diabetic neuropathy group and (ii) a control group. The diabetic neuropathy group was defined according to published reports and the following criteria^20-22^: (i) diagnosis of type 2 diabetes mellitus prior to the neurological symptoms, (ii) length-dependent symmetric sensory symptoms, (iii) abnormal neurological examination on thermal tests and (iv) lack of other known aetiologies leading to neuropathy, as determined by detailed history taking (such as prior exposure to neurotoxic agents) and laboratory evaluations including thyroid function tests, erythrocyte sedimentation rate, C-reactive protein, antinuclear antibody, extractable nuclear antigen, serum protein electrophoresis, vitamin B12, folic acid and homocysteine levels. The age- and sex-matched participants in the control group were free of neurological symptoms and demonstrated normal findings on neurological examinations and thermal thresholds without evidence of metabolic or endocrine disorders (such as diabetes mellitus), chronic infection, rheumatological disorder, malnutrition or toxin exposure based on the same laboratory assessments.

Informed consent was obtained before all of the procedures were performed. All of the procedures performed in the studies involving human participants adhered to the ethical standards of the institutional and/or national research committee, along with the 1964 Helsinki declaration and its later amendments or comparable ethical standards.

### Skin biopsy

The skin biopsy procedure followed our established protocol which is consistent with international guideline^23^ after obtaining the inform consent, which was approved by the IRB. A skin sample (3 mm in diameter) was obtained via a biopsy punch from the lateral side of the distal leg located 10 cm above the lateral malleolus under 2% lidocaine local anaesthesia.^9^ No suturing was needed, and the wound was covered with a piece of gauze. Wound healing occurred for 7∼10 days (similar to the healing rate of a typical abrasion wound).

### Immunohistochemical staining and quantification of epidermal innervation

The skin tissues were sectioned with a microtome at a thickness of 50 μm and subsequently fixed with 4% paraformaldehyde in 0.1 M phosphate-buffered saline (PBS) (pH 7.4) for 48 h.^24^ The sections were then immunostained following established protocols; specifically, they were quenched with 1% H_2_O_2_, blocked with 5% normal goat serum and incubated with rabbit antiserum to a neuron-specific marker known as protein gene product 9.5 (PGP9.5; 1:1000; Cedarlane, Burlington, NC) in 1% normal serum/Tris at 4°C for 24 h. On the next day, the sections were rinsed with Tris and further incubated with biotinylated goat anti-rabbit immunoglobulin G, after which they were incubated with the avidin-biotin complex (Vector, Burlingame, CA) and the chromogen known as SG (Vector).

The quantification of epidermal innervation followed the established protocol.^25^ Under 40× magnification with an Olympus BX40 microscope (Tokyo, Japan), the IENFs stained via PGP9.5 were counted throughout the entire section. The individual nerve fibres demonstrating branching points within the epidermis were counted as single fibres. For skin nerve fibres exhibiting branching points located in the dermis, each distinct nerve fibre was separately counted. Using Image-Pro PLUS software (Media Cybernetics, Silver Spring, MD), we measured the length of the epidermis along the upper margin of the stratum corneum.

The IENFd was defined as the number of nerve fibres divided by the length of the epidermis and is expressed in fibres per millimetre (fibres/mm). As IENFd declines with advancing age, establishing age-specific reference values is essential to avoid overdiagnosis of small-fibre neuropathy in older individuals and underdiagnosis in younger populations. In our laboratory, the cut-off value for IENFd at the distal leg was 5.88 fibres/mm for individuals younger than 60 years of age and 2.50 fibres/mm for those older than 60 years. This value represents the 5th percentile calculated from skin biopsy data obtained from normal subjects in our laboratory. Skin denervation was defined as an IENF below the age-specific cut-off value.

### Immunofluorescence staining

With the same fixation protocol that used for immunohistochemical staining, the skin samples were subjected to double immunofluorescence staining for PGP9.5 and Na-K-Cl cotransporter 1 (NKCC1).^26^ NKCC1 was used to label the epidermis. Instead of using collagen IV to label the basement membrane, we used NKCC1 to capture the entire epidermal layer for quantifying the epidermal area. Given that the epidermis is approximately rectangular with mild curvature and has a thickness much smaller than its length, the perimeter may serve as an approximate estimate of the epidermal surface length together with the dermal–epidermal junction length. After blocking with 0.1% Triton Milk for 1 h, the sections were incubated with mouse NKCC1 antiserum (clone T4, 1:150; Developmental Studies Hybridoma Bank, Iowa City, IA) and rabbit PGP9.5 anti-serum (CL7756AP, 1:1000; Cedarlane, Burlington, NC) at 4°C for 24 h. After being rinsed with Tris the next day, the sections were incubated with Alexa Fluor® 488 donkey anti-rabbit IgG (H + L) (711-545-152; Jackson ImmunoResearch, Inc., West Grove, PA) and Alexa Fluor® 647 donkey anti-mouse IgG (H + L) (715-605-151; Jackson ImmunoResearch, Inc., West Grove, PA) to label the nerve fibres and epidermis, respectively. After another rinse with Tris, the skin sections were then mounted with 2% n-propyl gallate in 60% glycerol. The sections were observed via a Zeiss AxioImagerM1 microscope. The Alexa Fluor 647 signals (for the epidermis) and Alexa Fluor 488 signals (for the nerve fibres) were imaged after excitation at wavelengths of 495 and 650 nm, respectively, via a light-emitting diode. The entire skin biopsy section was scanned, and the acquired images were then merged for colocalization of the epidermis and the IENFs for further quantification, as described in the following sections.

### Machine learning–based automatic quantification

**Figure 1:**
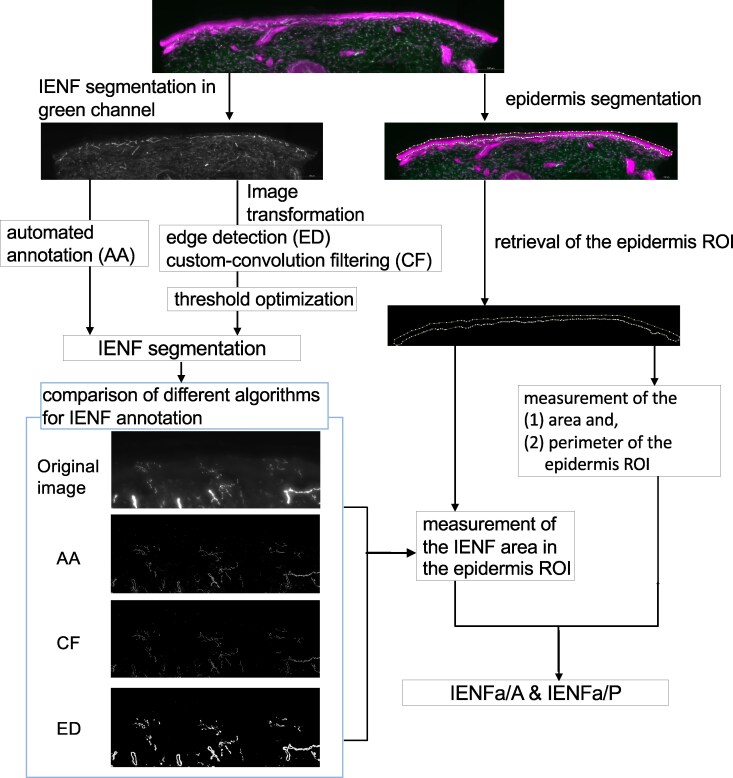
**Segmentation algorithm for the IENFs and epidermis.** The algorithm to develop IENFa biomarkers was divided into individual IENF and epidermis segmentation. The IENF segmentation was based on the green channel (PGP9.5). Three algorithms (AA, ED and CF) were established for IENF segmentation. The ED- and CF-transformed images were manually optimized based on thresholds before IENF segmentation. Comparisons between the original images and the three IENF segmented images are shown in the left lower corner. The epidermis was manually mapped on the merged images. The results of the IENFa based on the three algorithms were then divided by the area or perimeter of the epidermis to calculate the IENFa/A or IENFa/P, respectively. AA, automated annotation; CF, custom-convolution filtering; ED, edge detection; ROI, region of interest.

The machine learning–based automatic quantification process included two steps (Fig. 1): (i) the segmentation of the epidermis and (ii) the segmentation of the IENFs based on immunofluorescence images of the skin sections.

### Image processing: epidermis segmentation and feature retraction

Image processing was performed by using FIJI software (based on ImageJ 1.54i, NIH, Bethesda, MD) under Java 1.8.0_322 (64-bit). The epidermis was manually segmented by using the ‘polygon selections’ tool in a merged channel consisting of PGP9.5 (green) and NKCC1 (magenta). The scale was calibrated to 1.5 μm per pixel. The upper border of the epidermis excluded the stratum corneum, which did not contain small fibres and usually appeared to be translucent or slightly coloured.^27,28^ The lower border of the epidermis was defined along the rete ridge. The subepidermal plexus was excluded and used as a reference for the lower border, given that the plexus was located immediately below the epidermis. Hair follicles and sweat glands were excluded. If the hair follicles overlapped with the epidermis, their crossing points formed a virtual border of the epidermis. The perimeter and area of the epidermis segmentation were retrieved, and the mask was saved for subsequent measurement of the IENFa.

### Workflow for intraepidermal nerve fibre segmentation and area quantification

The definition and labelling of each IENF was based on the PGP9.5 single channel, termed as segmentation of IENF in the imaging-processing field. This study applied one machine learning–based algorithm: Weka-based automated annotation (AA) in ImageJ software to segment IENFs. AA directly labels nerve fibres on the original images using supervised pixel-level classification, i.e. to achieve correct labelling of IENFs through repeated feedback and practice. Detailed workflows are provided in the Supplementary graphical demonstration. The AA-achieved quantification was then compared with two conventional imaging-analysis tools to segment IENFs: edge detection (ED) and custom-convolution filtering (CF). ED and CF enhance nerve fibre signals through predefined image transformations according to the intensity gradients (in ED) or a matrix (termed as convolution kernel) to extract the IENF features from the images (in CF), respectively.

1. Edge detection: In ImageJ, two convolution methods were applied to enhance the contrast between the small nerve fibres and the background, including built-in ‘Edge detection’ and CF (Supplementary material).

The ‘Edge detection’ approach utilized the Sobel operator to enhance the edge. After the transformed images were acquired, threshold optimization was manually performed to determine the sampled area, followed by binarization via conversion to a mask with a black background. Further refinement, including the use of the morphological ‘Close’ and ‘Fill Holes’ approaches, was applied to obtain the segmented nerve fibre images.

2. Custom-convolution filtering: In addition to the ‘Edge detection’ approach, we tested several convolution kernels and determined the kernel being used based on our subjective judgment (Supplementary Table 1). The workflow following the acquisition of the convolutionized images was similar to that utilized for ‘Edge detection’.
3. Weka-based AA: We applied the built-in Weka trainable segmentation package in ImageJ for AA.^29^ A prototype classifier was initially trained using two single representative skin biopsy images from one subject (https://reurl.cc/GN1X8D). We applied the trained model to the same PGP9.5 single-channel images as described above. After the initial segmented image was obtained using the trained model, further adjustments were conducted by providing the information that the segmented pixels were classified either correctly or incorrectly inside of the Weka trainable segmentation package. The final segmented nerve fibre images were saved for further analysis (Supplementary: graphic demonstration of IENF segmentation).

As a result, AA was used to directly label the small nerve fibres on the original images, whereas CF and ED were utilized to transform the images to contrast the small nerve fibres against the background. The segmented nerve fibre images were then analysed with the predefined epidermis mask. In our preliminary study, the particles smaller than 0.40 μm^2^ were difficult to differentiate from the background noise. As a result, particles smaller than 0.40 μm^2^ were excluded from analysis. The particles located at the periphery of the epidermis mask were also excluded. The total area of the IENF particles was retrieved as the IENFa. Two parameters were derived by normalizing the IENFa to the length of the epidermis perimeter (IENFa/P) or the epidermis area (IENFa/A). Thus, six IENFa parameters were ultimately determined: (i) the IENFa/P derived from ED, CF and AA and (ii) the IENFa/A derived from ED, CF and AA. Each subject had two skin sections, and the average of the IENFa parameters was defined as the IENFa/A or IENFa/P for the subject.

All intra-rater and inter-rater ICCs were excellent for IENFa/A, IENFa/P and epidermis parameters. The intra-rater and inter-rater ICCs of IENFa/A-AA and IENFa/P-AA were significantly higher than those of ED and CF, as determined by the cluster bootstrap method with pairwise comparisons and Holm–Bonferroni correction (Supplementary Table 2).

### Nerve conduction studies

Nerve conduction studies and electromyograms were performed by using a Viking IV Electromyographer (Nicolet, Madison, WI) in all of the patients following established methods. The bilateral sural nerves and bilateral tibial nerves were assessed. The mean sural sensory nerve action potentials (SNAPs) and tibial compound muscle action potentials (CMAPs) were calculated for further analysis following the consensus and our published report.^12,20,30,31^

### Psychophysical measures of the thermal threshold

Psychophysical assessment of thermal thresholds was performed using a Thermal Sensory Analyzer (Medoc Advanced Medical Systems, Minneapolis, MN), following procedures described previously.^32^ Briefly, the device delivers thermal stimuli of predefined intensity determined by an automated algorithm. The stimulus intensity is adjusted in fixed increments or decrements based on the participant’s response (i.e. perception or non-perception of the stimulus). Using this adaptive method, sensory thresholds for warm and cold modalities were determined at the foot and toe.

### Statistical analysis

Continuous variables, which are expressed as the means ± SDs, were compared by using the *t*-test if they followed a normal distribution (via the Shapiro–Wilk test). A non-parametric test was used if the variables did not follow a normal distribution. The *χ*² test or Fisher’s exact test was used to compare the categorical data. Inter-rater and intra-rater reliabilities were assessed by using intraclass correlation coefficients (ICCs). To evaluate uncertainty, we applied non-parametric bootstrap resampling with 1000 replicates at the subject level. For each replicate, the ICC estimates were recalculated across the biomarkers. Pairwise differences between the biomarkers were subsequently obtained by subtracting bootstrap distributions, and 95% confidence intervals were derived from the percentile method.^33,34^ Significance was defined when the confidence interval of the difference did not cross zero, with *P* values being adjusted for multiple comparisons by using the Holm method. We used univariate and multivariate linear regression analyses to examine the contribution of the independent variables to the dependent variable. To compare the strengths of the dependent correlations sharing one variable (such as the correlation between IENF parameters and age), the Steiger’s Z test was performed.^35^ Pearson correlation analysis was used to assess the association between IENFa and IENFd. Receiver operating characteristic (ROC) analysis was conducted to examine the diagnostic performance of small-fibre neuropathy. The Youden’s index was calculated as the sensitivity plus specificity minus 1. The threshold of the IENFa was determined based on the largest Youden’s index. The Delong’s test was used to determine the significant difference between the area under the curve among the different IENFa parameters. All of the analyses were performed by using R studio software [version 4.4.1; R Core Team (2024-06-14)]. The code was available in the following link: https://drive.google.com/file/d/1lPuyZicXxj_jl3CVZLmKtYm6lRVzZ5D8/view?usp=sharing). The results were considered to be significant at a *P* value of <0.05.

## Results

### Demographic profiles of the recruited subjects

In this study, 48 patients with diabetic neuropathy (male/female: 31/17; aged 59.3 ± 12.5 years; Table 1) were selected based on the clinical manifestations of symmetric and length-dependent sensory symptoms with impaired thermal function on neurological examinations. In addition to diabetes, some patients also demonstrated metabolic derangements according to blood biochemical tests. Chronic kidney disease was present in approximately half of the patients (51.2%), while 34.5% had hyperlipidaemia. To establish normative values and compare them with those of diabetic patients, we recruited 63 control subjects (male/female: 40/23; aged 56.8 ± 13.0 years; Table 2). Overall, the IENFd and area-based morphometry of the IENFa were significantly greater in the control group than in the diabetic neuropathy group (with unadjusted and age- and sex-adjusted *P* < 0.001) (Table 2).

**Table 1:** Demographic, anthropometric and biochemical profiles of patients with diabetic neuropathy

	Diabetic patients (*n* = 48)
Age (years)	59.3 ± 12.5 (24.8–82.9)
Sex (male/female)	31/17
Diabetic duration (years)	9.7 ± 8.7 (0.1–40)
HbA1c (%)	7.62 ± 2.15 (5–15.1)
Fasting glucose (mg/dL)	131.2 ± 50.0 (53–256)
Body height (cm)	166.4 ± 9.0 (148–181)
Body weight (kg)	65.9 ± 13.3 (43.5–100)
Body mass index (kg/m^2^)	23.7 ± 3.9 (15.9–34)
Blood urea nitrogen (mg/dL)	23.4 ± 18.3 (9.9–104.3)
Creatinine (mg/dL)	1.41 ± 1.59 (0.5–7.8)
Total cholesterol (mg/dL)	195.9 ± 58.0 (67–321)
Low-density lipoprotein (mg/dL)	108.6 ± 37.1 (44–200)
High-density lipoprotein (mg/dL)	43.5 ± 16.0 (25–107)
Triglyceride (mg/dL)	156.2 ± 117.2 (40–558)
Tibial CMAP (mV)	11.2 ± 5.3 (0.5–21)
Sural SNAP (µV)	7.5 ± 5.5 (0–16.5)
Warm threshold at the foot (s)	40.4 ± 3.9 (34.7–50)
Warm threshold at the toe (s)	43.7 ± 4.3 (35–50)
Cold threshold at the foot (s)	29.1 ± 4.1 (0–31.3)
Cold threshold at the toe (s)	24.9 ± 8.5 (0–31.0)

The values are expressed as the mean ± SD (range).

CMAP, compound muscle action potential; SNAP, sensory nerve action potential.

**Table 2:** Comparison of demographic and IENF measurements between the control and neuropathy groups

	Neuropathy (*n* = 48)^a^	Control (*n* = 63)^a^	*P* value	Adjusted *P* value^b^
Age	59.3 ± 12.5	56.8 ± 13.0	0.296	
Male sex	31 (65%)	40 (63%)	0.906	
IENFd	1.58 ± 1.98 (1.01–2.16)	7.83 ± 2.94 (7.04–8.28)	<0.001	<0.001
Area-based IENFa (IENFa/A) (100*µm^2^/µm^2^)				
IENFa/A (AA)	0.35 ± 0.39 (0.24–0.47)	1.02 ± 0.49 (0.94–1.16)	<0.001	<0.001
IENFa/A (CF)	0.32 ± 0.32 (0.23–0.42)	0.91 ± 0.48 (0.81–1.02)	<0.001	<0.001
IENFa/A (ED)	0.61 ± 0.62 (0.43–0.79)	2.11 ± 1.18 (1.82–2.32)	<0.001	<0.001
Length-based IENFa (IENFa/P) (µm^2^/µm)				
IENFa/P (AA)	0.10 ± 0.11 (0.07–0.14)	0.32 ± 0.15 (0.30–0.37)	<0.001	<0.001
IENFa/P (CF)	0.09 ± 0.09 (0.07–0.12)	0.29 ± 0.15 (0.26–0.32)	<0.001	<0.001
IENFa/P (ED)	0.18 ± 0.20 (0.13–0.24)	0.67 ± 0.36 (0.58–0.74)	<0.001	<0.001

A, area of the epidermis; P, perimeter of the epidermis; AA, automated annotation; CF, custom-convolution filtering; ED, edge detection.

^a^Mean ± SD (95% confidence interval); *n* (%).

^b^Adjusted for age and sex via multivariate linear regression.

### The nature of intraepidermal nerve fibre area and its correlations to intraepidermal nerve fibre density

**Figure 2:**
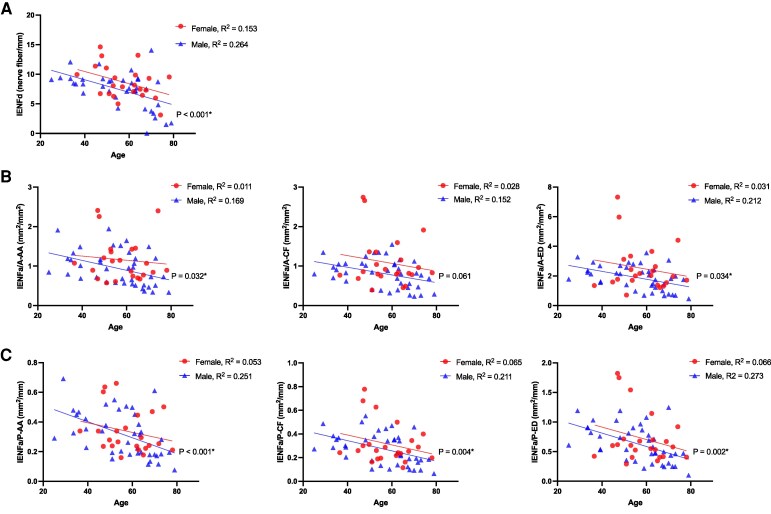
**Correlation of IENFa with age in the control group.** Scatter plots illustrate the correlation between age and various nerve fibre metrics in the control group. Each data point represents an individual subject. (**A**) Correlation between IENFd and age. (**B**) Correlation between IENFa/A and age using three quantification methods (AA, CF and ED). (**C**) Correlation between IENFa/P and age. Solid lines represent the fitted linear regression models for female (red) and male (blue) subjects. The coefficient of determination () and statistical significance (-value) are provided for each regression analysis. *P < 0.05, by the linear regression analysis; IENFa/P, intraepidermal nerve fibre area versus epidermis length; IENFa/A, intraepidermal nerve fibre area versus epidermis area; IENFd, intraepidermal nerve fibre density; AA, automated annotation; CF, custom-convolution filtering; ED, edge detection; IENFa/A, IENF area normalized by epidermis area (mm^2^/mm^2^); IENFa/P, IENF area normalized by epidermis length (mm^2^/mm).

This study subsequently examined the relationships of IENFd and IENFa with age and sex in the control group after the establishment of the platform of nerve area-based morphometry. Both IENFd and IENFa showed significant inverse associations with age in the control group (Fig. 2).

In multivariate models, IENFd decreased by approximately 0.10 fibres/mm^2^ per year (*β* = −0.105, 95% CI −0.156∼−0.055; *P* < 0.001), while representative area-based and length-based IENFa metrics showed comparable age-related declines (*β* range −0.01 to −0.03; all *P* < 0.05) (Supplementary Table 3). In the univariate analysis, after adjustment for age, only IENFd and CF-based IENFa/A showed significant sex dependence, with females exhibiting higher values than males (IENFd: *β* = −1.45, 95% CI −2.81 to −0.09; *P* < 0.05) (Supplementary Table 3), whereas other IENFa metrics were not significantly associated with sex. In the multivariate analyses, all of the parameters (including IENFd, IENFa/A and IENFa/P) were inversely correlated with age, and only the IENFd and IENFa/A-CF were determined to be sex dependent. Notably, after the IENF parameters and age were standardized, length-based metrics (IENFd and IENFa/P) demonstrated stronger inverse correlations with age than area-based metrics (IENFa/A). For example, standardized IENFd showed a robust age association (*β* = −0.46; *P* < 0.001), whereas standardized IENFa/A exhibited more modest effects (*β* ≈ −0.26 to −0.29; *P* < 0.05). This difference was statistically confirmed using Steiger’s Z test (Supplementary Table 3).

**Figure 3:**
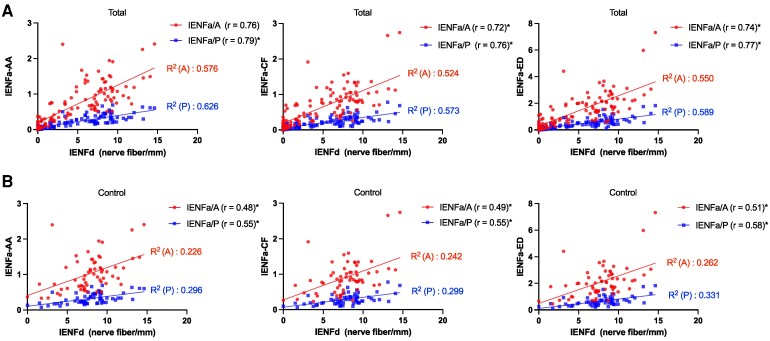
**Correlation of IENFa with IENFd.** Scatter plots illustrate the Pearson correlations between IENFd and three IENFa quantification approaches: AA, CF and ED. Each data point represents an individual subject. (**A**) Correlations in the total cohort (*n* = 111), including patients with neuropathy (*n* = 48) and healthy controls (*n* = 63). (**B**) Correlations within the control group (*n* = 63). Solid lines represent the fitted linear regression models. Pearson correlation coefficients (*r*) are provided in the plot legend, and coefficients of determination (*R*^2^) are colour-coded to correspond to their respective regression lines (red for IENFa/A and blue for IENFa/P). *P < 0.05, by the Pearson correlation analysis; IENFa/P, intraepidermal nerve fibre area versus epidermis length; IENFa/A, intraepidermal nerve fibre area versus epidermis area; IENFd, intraepidermal nerve fibre density; AA, automated annotation; CF, custom-convolution filtering; ED, edge detection; IENFa/P, IENF area normalized by epidermis length (mm); IENFa/A, IENF area normalized by epidermis area (ratio).

We then explored the relationship between the IENFa and IENFd. Among all of the subjects, the IENFa/P and the IENFa/A were strongly correlated with the IENFd (Pearson correlation coefficient > 0.7; *P* < 0.05) (Fig. 3). This study further analysed this relationship in the control group, and a moderate correlation was detected (Pearson correlation coefficient: 0.5∼0.6; *P* < 0.05). Additionally, the coefficients of variation were lower for the AA-based IENFa/A (AA: 47.6%, CF: 56.0% and ED: 52.9%) and IENFa/P (AA: 46.5%, CF: 53.7% and ED: 51.6%).

### Diagnostic performance of small-fibre sensory neuropathy

**Figure 4:**
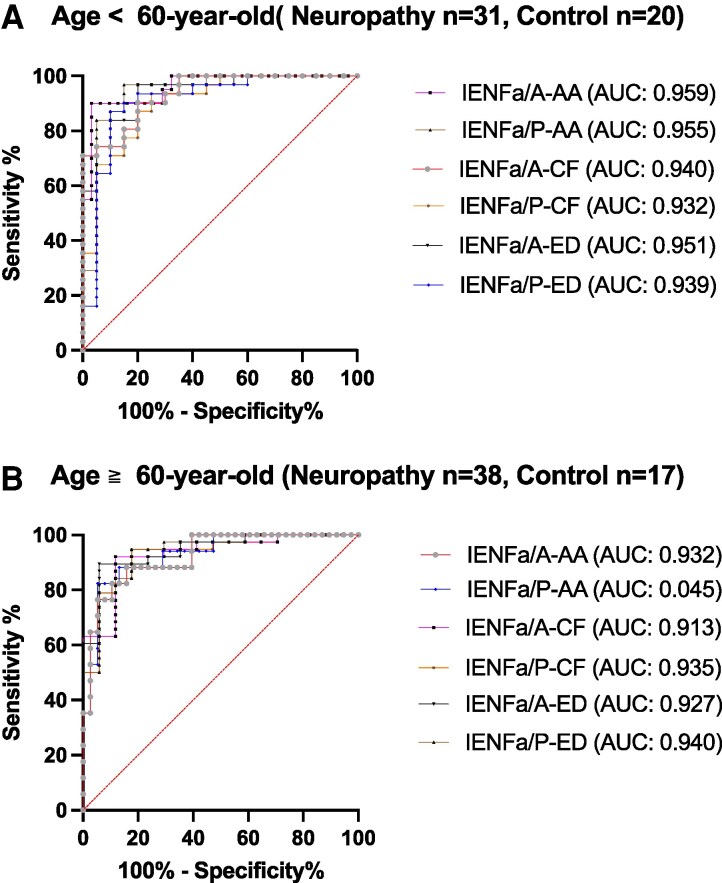
**Diagnostic performance of IENFa in diagnosing IENFd-defined small-fibre neuropathy.** Receiver operating characteristic analyses were performed in the different age groups (age < 60 years old in **A** and age ≧ 60 years old in **B**). Small-fibre neuropathy cases were defined as patients with type 2 diabetes mellitus presenting length-dependent symmetric sensory symptoms, abnormal thermal sensory testing and exclusion of other causes of neuropathy based on clinical and laboratory evaluations. Control participants were age- and sex-matched individuals without neurological symptoms, with normal neurological examinations and thermal thresholds and without metabolic, endocrine, infectious, rheumatologic, nutritional or toxic aetiologies. All of the IENFa/A and IENFa/P biomarkers demonstrated equivalent and good performance among the different age groups. The AUC values were shown in the corresponding annotations for each IENFa parameter. IENFa/P, intraepidermal nerve fibre area versus epidermis length; IENFa/A, intraepidermal nerve fibre area versus epidermis area; IENFd, intraepidermal nerve fibre density; AA, automated annotation; CF, custom-convolution filtering; ED, edge detection; AUC, area under the curve.

To investigate the diagnostic performance of IENFa, we used the ROC analysis to determine the cut-off value of IENFa. The criteria for small-fibre sensory neuropathy included typical symptoms, clinical signs and reduced IENFd. Due to the fact that the cut-off value of IENFd differed between the groups of the subjects aged ≥60 years and the younger subjects according to our normative database,^9,12,20^ we investigated the threshold of IENFa in these two groups, as well as in the overall cohort (Supplementary Table 4; Fig. 4). Overall, the Youden’s index values were determined to be between 0.7 and 0.87 (indicating good to very good performance), and the AUC value was approximately 0.95 (indicating excellent performance). Applying age-specific reference values resulted in improved diagnostic performance among subjects aged <60 years relative to the overall cohort. In summary, the diagnostic performance of small-fibre neuropathy was comparable among the different IENFa approaches. Thus, we used only the most intuitive and genuine approaches (IENFa/P-AA and IENFa/A-AA) in the following analysis.

### Associations between small-fibre nerve fibre metrics and large-fibre nerve fibre function in diabetic neuropathy

In addition to the diagnosis of small-fibre neuropathy, we explored the clinical significance of the IENFa-based quantification system by examining biochemical and electrophysiological correlations. In the majority of diabetic neuropathy cases, the initial manifestations include symptoms and signs of small-diameter nerve fibres with gradual involvement of large-diameter nerve fibres, which is demonstrated by small-fibre nerve degeneration in parallel with large-fibre nerve impairment as the disease progresses. As a result, we examined the relationships between the IENF parameters and the parameters of the electrophysiological studies (Table 3). In summary, most of the IENFd and IENFa biomarkers (except for IENFa/P-ED) were significantly correlated with sural SNAP amplitudes. Interestingly, only the IENFa/P-AA and IENFa/A-AA biomarkers were significantly correlated with the tibial CMAP amplitude. The correlations with biochemical profiles (including HbA1c, fasting glucose, duration of diabetes and lipid profiles) were not significant for either IENFd or IENFa.

**Table 3:** Electrophysiological correlation to IENFd and IENFa in diabetic neuropathy

	Sural SNAP amplitudes	Tibial CMAP amplitudes
IENFd	0.62 (0.23, 0.84)**	0.42 (−0.05, 0.73)
IENFa/A		
IENFa/A-AA	0.60 (0.20, 0.83)**	0.50 (0.05, 0.78)*
IENFa/A-CF	0.54 (0.12, 0.80)*	0.45 (−0.01, 0.75)
IENFa/A-ED	0.51 (0.07, 0.78)*	0.43 (−0.03, 0.74)
IENFa/P		
IENFa/P-AA	0.55 (0.13, 0.80)*	0.49 (0.04, 0.77)*
IENFa/P-CF	0.46 (0.002, 0.75)*	0.43 (−0.03, 0.74)
IENFa/P-ED	0.43 (−0.04, 0.74)	0.39 (−0.08, 0.72)

AA, automated annotation; CF, custom-convolution filtering; ED, edge detection; SNAP, sensory nerve action potential; CMAP, compound muscle action potential.

^*^<0.05, **<0.01, ***<0.001.

## Discussion

In this study, new IENFa-based biomarkers for the automatic quantification of skin innervation in diabetic neuropathy (IENFa/A and IENFa/P) were developed by applying a machine learning algorithm to quantify IENFs and the epidermis. We further characterized these newly established IENF parameters with two major features: (i) comparable diagnostic performance with the gold standard of small-fibre neuropathy (IENFd) and (ii) concurrent degeneration of small and large sensory nerves in diabetic neuropathy.

This study not only developed two IENFa parameters (IENFa/P and IENFa/A) but also demonstrated their clinical applications and significance. These two biomarkers shared similar features with IENFd, although they exhibited a minor difference. All three indices (IENFd, IENFa/A and IENFa/P) were correlated with age.^36^ In summary, IENFd, IENFa/A-CF and IENFa/A-ED were dependent on age and sex, with IENFa/A-AA and IENFa/P being solely dependent on age. Compared with the IENFd and IENFa/P biomarkers, the IENFa/A biomarker demonstrated a significantly lower degree of correlation with age. These findings suggest that each IENF parameter exhibits unique characteristics in different clinical scenarios.

Currently, IENFd is the gold standard for the diagnosis of small-fibre neuropathy.^6-8^ Thus, this study compared the diagnostic performance between the IENFa/A and IENFa/P biomarkers. The AUC value was determined to be approximately 0.95, and there was no significant difference observed between the IENFa parameters in the diagnosis of IENFd-defined small-fibre neuropathy. The analysis demonstrated that the diagnostic performance for small-fibre neuropathy was comparable between IENFa and IENFd. Furthermore, the AUC values ranged from 0.90 to 0.95. These observations indicate the equivalent diagnostic performance between the IENFa and IENFd biomarkers. Moreover, similar to the age-specific cut-off values used for IENFd, age-specific cut-off values for IENFa parameters should also be implemented in clinical practice. Our data demonstrate that the use of age-specific cut-off values improves diagnostic performance in subjects younger than 60 years. However, the definition of small-fibre neuropathy currently relies primarily on IENFd, which is considered the gold standard. This may result in a cohort enriched for small-fibre neuropathy. Consequently, the ROC analyses primarily reflect the concordance between IENFa and IENFd rather than the independent diagnostic performance of IENFa. Future studies in independent cohorts are warranted to validate the independent diagnostic utility of IENFa. As a result, IENFa could be an alternative diagnostic biomarker for small-fibre neuropathy. Furthermore, this area-based parameter of IENFa also implies global axonal atrophy, which is complementary to the skin nerve degeneration index of IENFd.

Diabetic neuropathy mainly affects small-diameter sensory nerves at the early stage. When the severity of the condition increases, the large-diameter motor nerve fibres are also affected as a result of disease progression.^1,2,37^ This progression likely reflects the cumulative effects of metabolic stress, microvascular dysfunction and axonal injury across different nerve fibre types, rather than isolated fibre-specific pathology. Accordingly, the observed correlations between IENFa metrics and sural SNAP amplitudes suggest that IENFa captures not only small-fibre pathology but also the overall burden of peripheral nerve involvement. Notably, the association between the IENFa-AA metric and tibial CMAP amplitudes may reflect more advanced, length-dependent axonal degeneration involving both sensory and motor fibres. This discrepancy again highlights the application of different IENF biomarkers according to clinical scenarios. Whether these associations solely represent generalized neuropathy severity or additionally involve shared pathophysiological mechanisms between small- and large-fibre degeneration cannot be fully disentangled in the current study and requires a prospective investigation. Nevertheless, the performance of these newly developed IENFa biomarkers was comparable to that of IENFd and further reflected the disease activity of diabetic neuropathy.

Traditionally, IENFd is defined via manual counting on immunostaining images.^7,8,38-40^ However, two issues are demonstrated regarding the use of IENFd: (i) the number versus the total area of IENFs and (ii) time efficiency. Theoretically, the total IENFa could differ despite the same IENFd being observed.^8,21,38,40^ However, the morphometric characteristics of IENFs have not yet been systemically explored, and new parameters of IENFa provide clinical significance, including gross axonal atrophy being observed in parallel with a reduction in IENFs. This study utilized double-labelling immunofluorescent imaging to separately delineate IENFs and the epidermis in different fluorochrome channels. Furthermore, we applied machine learning–based automatic segmentation to quantify the IENFs with various computer vision analysis algorithms, including AA, ED and CF. All of these methods demonstrated similar diagnostic efficacy for IENFd-defined small-fibre neuropathy. Among these algorithms, automatic annotation was observed to be the most intuitive, with the highest intra-rater and inter-rater reliabilities being determined; moreover, this method was free of distortion in labelling IENFs, and the remaining two methods required transformation to a certain degree. Thus, the AA-based algorithm is the preferred method for the future quantification of IENFa.

The current study has several limitations. First, this study focused on diabetic neuropathic features but did not investigate general neuropathic entities. Further investigations on general neuropathic examinations, such as psychophysical measures of the thermal threshold, are warranted to evaluate the clinical application and external validation of the IENFa. Second, we did not directly measure the basement membrane length, as is done for traditional IENFd. Because the epidermis is approximately rectangular with mild curvature and has a thickness much smaller than its length, the epidermal perimeter can be regarded as an approximation of the combined epidermal surface length and dermal–epidermal junction length. Accordingly, the IENFa-to-perimeter ratio (IENFa/P) is conceptually comparable to the conventional IENFd. We acknowledge that the perimeter is not identical to the true dermal–epidermal junction length. Accurate delineation of the dermal–epidermal junction from the segmented epidermis would require more advanced computer vision–based analytical approaches. Third, we did not perform a direct comparison of IENFd measured by immunohistochemistry and immunofluorescence. We acknowledge that immunohistochemistry may underestimate IENFd compared with immunofluorescence. However, our primary aim was not to compare absolute values between staining methods. Instead, we focused on examining the relationship between IHC-derived IENFd and immunofluorescence-based IENFa parameters, under the assumption that relative trends would be preserved across methods.^41^ Fourth, Particles smaller than 0.40 µm^2^ were excluded from the analysis to reduce noise and improve segmentation robustness. However, this threshold may have resulted in the exclusion of atrophic or fragmented IENFs, which are commonly observed in diabetic neuropathy, potentially leading to an underestimation of nerve fibre pathology. Further study with higher numerical aperture would solve this issue by reducing background noise. Fourth, particles smaller than 0.40 µm^2^ were excluded from the analysis to reduce noise and improve segmentation robustness. However, this threshold may result in the exclusion of atrophic or fragmented IENFs, which are commonly observed in diabetic neuropathy, potentially leading to an underestimation of nerve fibre pathology. Further study with higher numerical aperture would solve this issue by reducing background noise.^42^ Last, we did not demonstrate correlations between the biochemical profiles of diabetic patients and lipid profiles, which may be related to the limited sample size.^43^ Further prospective cohort studies with regular glucose monitoring are needed to investigate the effects of cumulative exposure to hyperglycaemia on skin nerve degeneration. Nevertheless, this machine learning–based automatic quantification method offers technical advancements in terms of time-saving effects, consistency and high reliability for different measures of skin innervation.

## Conclusion

This study established an area-based biomarker of IENFa for the automatic quantification of skin innervation. The diagnostic accuracy of IENFa was comparable and equivalent to the current standard of IENFd. Moreover, the correlations of IENFa with the parameters for the degeneration of large-fibre nerves provide compelling evidence for parallel involvement among nerve fibres of different modalities in diabetic neuropathy.

## Supplementary Material

fcag113_Supplementary_Data

## Data Availability

The data that support the findings of this study contain patient-level clinical and histopathological information and are therefore not publicly available due to privacy and ethical restrictions. Access to the data may be considered upon reasonable request and approval by the corresponding institutional review board. The image analysis codes and scripts used in this study are publicly available in the Supplementary material and at the following URL: https://drive.google.com/file/d/1lPuyZicXxj_jl3CVZLmKtYm6lRVzZ5D8/view?usp=sharing.

## References

[1] Feldman EL, Callaghan BC, Pop-Busui R, et al Diabetic neuropathy. Nat Rev Dis Primers. 2019;5:41. 31197153 10.1038/s41572-019-0092-1

[2] Pop-Busui R, Boulton AJ, Feldman EL, et al Diabetic neuropathy: A position statement by the American diabetes association. Diabetes Care. 2017;40:136–154. 27999003 10.2337/dc16-2042 PMC6977405

[3] Borbjerg MK, Wegeberg A-M, Nikontovic A, et al Understanding the impact of diabetic peripheral neuropathy and neuropathic pain on quality of life and mental health in 6,960 people with diabetes. Diabetes Care. 2025;48:588–595. 39932781 10.2337/dc24-2287

[4] Ebenezer G, Polydefkis M. Epidermal innervation in diabetes. Handb Clin Neurol. 2014;126:261–274. 25410228 10.1016/B978-0-444-53480-4.00020-5

[5] Sloan G, Donatien P, Privitera R, et al Vascular and nerve biomarkers in thigh skin biopsies differentiate painful from painless diabetic peripheral neuropathy. Front Pain Res (Lausanne). 2024;5:1485420. 39512388 10.3389/fpain.2024.1485420 PMC11543357

[6] Devigili G, Rinaldo S, Lombardi R, et al Diagnostic criteria for small fibre neuropathy in clinical practice and research. Brain. 2019;142:3728–3736. 31665231 10.1093/brain/awz333 PMC6906595

[7] Egenolf N, Zu Altenschildesche CM, Kreß L, et al Diagnosing small fiber neuropathy in clinical practice: A deep phenotyping study. Ther Adv Neurol Disord. 2021;14:17562864211004318. 34335876 10.1177/17562864211004318 PMC8283814

[8] Haroutounian S, Todorovic MS, Leinders M, et al Diagnostic criteria for idiopathic small fiber neuropathy: A systematic review. Muscle Nerve. 2021;63:170–177. 32989823 10.1002/mus.27070

[9] Chien HF, Tseng TJ, Lin WM, et al Quantitative pathology of cutaneous nerve terminal degeneration in the human skin. Acta Neuropathol. 2001;102:455–461. 11699558 10.1007/s004010100397

[10] Freeman R, Gewandter JS, Faber CG, et al Idiopathic distal sensory polyneuropathy: ACTTION diagnostic criteria. Neurology. 2020;95:1005–1014. 33055271 10.1212/WNL.0000000000010988 PMC7734920

[11] Khoshnoodi MA, Truelove S, Burakgazi A, Hoke A, Mammen AL, Polydefkis M. Longitudinal assessment of small fiber neuropathy: Evidence of a non-length-dependent distal axonopathy. JAMA Neurol. 2016;73:684–690. 27065313 10.1001/jamaneurol.2016.0057

[12] Chao C-C, Hsueh H-W, Kan H-W, et al Skin nerve pathology: Biomarkers of premanifest and manifest amyloid neuropathy. Ann Neurol. 2019;85:560–573. 30737830 10.1002/ana.25433

[13] Lauria G, Faber CG, Cornblath DR. Skin biopsy and small fibre neuropathies: Facts and thoughts 30 years later. J Neurol Neurosurg Psychiatry. 2022;93:915–918. 35246491 10.1136/jnnp-2021-327742 PMC9380509

[14] Friedrich MU, Relton S, Wong D, Alty J. Computer vision in clinical neurology: A review. JAMA Neurol. 2025;82:407–415. 10.1001/jamaneurol.2024.5326 39960732

[15] Wang D, Honnorat N, Toledo JB, et al Deep learning reveals pathology-confirmed neuroimaging signatures in Alzheimer's, vascular and Lewy body dementias. Brain. 2025;148:1963–1977. 39657969 10.1093/brain/awae388 PMC12129746

[16] Ruan Y, Bellot A, Moysova Z, et al Predicting the risk of inpatient hypoglycemia with machine learning using electronic health records. Diabetes Care. 2020;43:1504–1511. 32350021 10.2337/dc19-1743

[17] Lam C, Wong YL, Tang Z, et al Performance of artificial intelligence in detecting diabetic macular edema from Fundus photography and optical coherence tomography images: A systematic review and meta-analysis. Diabetes Care. 2024;47:304–319. 38241500 10.2337/dc23-0993

[18] Fortanier E, Hostin MA, Michel CP, et al Comparison of manual vs artificial intelligence-based muscle MRI segmentation for evaluating disease progression in patients with CMT1A. Neurology. 2024;103:e210013. 39447103 10.1212/WNL.0000000000210013

[19] Tveit J, Aurlien H, Plis S, et al Automated interpretation of clinical electroencephalograms using artificial intelligence. JAMA Neurol. 2023;80:805–812. 37338864 10.1001/jamaneurol.2023.1645 PMC10282956

[20] Chao CC, Tseng MT, Lin YJ, et al Pathophysiology of neuropathic pain in type 2 diabetes: Skin denervation and contact heat-evoked potentials. Diabetes Care. 2010;33:2654–2659. 20841612 10.2337/dc10-1135 PMC2992207

[21] Kan H-W, Hsieh J-H, Wang S-W, et al Nonpermissive skin environment impairs nerve regeneration in diabetes via Sec31a. Ann Neurol. 2022;91:821–833. 35285061 10.1002/ana.26347

[22] Tesfaye S, Boulton AJ, Dyck PJ, et al Diabetic neuropathies: Update on definitions, diagnostic criteria, estimation of severity, and treatments. Diabetes Care. 2010;33:2285–2293. 20876709 10.2337/dc10-1303 PMC2945176

[23] European federation of neurological societies/peripheral nerve society guideline on the use of skin biopsy in the diagnosis of small fiber neuropathy. Report of a joint task force of the European federation of neurological societies and the peripheral nerve society. J Peripher Nerv Syst 2010;15:79–92. 20626771 10.1111/j.1529-8027.2010.00269.x

[24] Hsieh ST, Lin WM. Modulation of keratinocyte proliferation by skin innervation. J Invest Dermatol. 1999;113:579–586. 10504444 10.1046/j.1523-1747.1999.00737.x

[25] Pan CL, Tseng TJ, Lin YH, Chiang MC, Lin WM, Hsieh ST. Cutaneous innervation in Guillain-Barre syndrome: Pathology and clinical correlations. Brain. 2003;126:386–397. 12538405 10.1093/brain/awg039

[26] Van Acker N, Ragé M, Sluydts E, et al Automated PGP9.5 immunofluorescence staining: A valuable tool in the assessment of small fiber neuropathy? BMC Res Notes. 2016;9:280. 27215701 10.1186/s13104-016-2085-4 PMC4878004

[27] Talagas M, Lebonvallet N, Leschiera R, Elies P, Marcorelles P, Misery L. Intra-epidermal nerve endings progress within keratinocyte cytoplasmic tunnels in normal human skin. Exp Dermatol. 2020;29:387–392. 32003039 10.1111/exd.14081

[28] Johansson O, Wang L, Hilliges M, Liang Y. Intraepidermal nerves in human skin: PGP 9.5 immunohistochemistry with special reference to the nerve density in skin from different body regions. J Peripher Nerv Syst. 1999;4:43–52. 10197064

[29] Arganda-Carreras I, Kaynig V, Rueden C, et al Trainable Weka Segmentation: A machine learning tool for microscopy pixel classification. Bioinformatics. 2017;33:2424–2426. 28369169 10.1093/bioinformatics/btx180

[30] Proceedings of a consensus development conference on standardized measures in diabetic neuropathy. Electrodiagnostic measures. Neurology 1992;42:1827–1829. 1513478

[31] Hsueh SJ, Lin CH, Lee NC, et al Unique clinical and electrophysiological features in the peripheral nerve system in patients with sialidosis—A case series study. Orphanet J Rare Dis. 2024;19:217. 38790028 10.1186/s13023-024-03216-8 PMC11127318

[32] Pan CL, Lin YH, Lin WM, Tai TY, Hsieh ST. Degeneration of nociceptive nerve terminals in human peripheral neuropathy. Neuroreport. 2001;12:787–792. 11277584 10.1097/00001756-200103260-00034

[33] Seydi MR, Pini A, Pataky TC, Schelin L. Confidence sets for intraclass correlation coefficients in test-retest curve measurements. J Biomech. 2024;173:112232. 39089220 10.1016/j.jbiomech.2024.112232

[34] Huang FL . Using cluster bootstrapping to analyze nested data with a few clusters. Educ Psychol Meas. 2018;78:297–318. 29795957 10.1177/0013164416678980 PMC5965657

[35] Zhang Q . Comparing methods for assessing a difference in correlations with dependent groups, measurement error, nonnormality, and incomplete data. Psychol Methods. 2024;29:767–788. 35901379 10.1037/met0000522

[36] Diedenhofen B, Musch J. Cocor: A comprehensive solution for the statistical comparison of correlations. PLoS One. 2015;10:e0121945. 25835001 10.1371/journal.pone.0121945 PMC4383486

[37] Savelieff MG, Feldman EL. Diabetic peripheral neuropathy: Predictors of disease progression. Neurology. 2024;103:e209705. 39008803 10.1212/WNL.0000000000209705 PMC13007812

[38] Devigili G, Tugnoli V, Penza P, et al The diagnostic criteria for small fibre neuropathy: From symptoms to neuropathology. Brain. 2008;131:1912–1925. 18524793 10.1093/brain/awn093 PMC2442424

[39] Devigili G, Marchi M, Lauria G. Small fiber neuropathy: Expanding diagnosis with unsettled etiology. Curr Opin Neurol. 2025;38:485–495. 40772640 10.1097/WCO.0000000000001418 PMC12419023

[40] Hsieh S-T, Anand P, Gibbons CH, Sommer C. Small fiber neuropathy and related syndromes: pain and neurodegeneration. Springer Singapore; 2019.

[41] Nolano M, Biasiotta A, Lombardi R, et al Epidermal innervation morphometry by immunofluorescence and bright-field microscopy. J Peripher Nerv Syst. 2015;20:387–391. 26309146 10.1111/jns.12146

[42] Li Y, Huang F. A statistical resolution measure of fluorescence microscopy with finite photons. Nat Commun. 2024;15:3760. 38704387 10.1038/s41467-024-48155-x PMC11069581

[43] Chao CC, Hsieh SC, Yang WS, et al Glycemic control is related to the severity of impaired thermal sensations in type 2 diabetes. Diabetes Metab Res Rev. 2007;23:612–620. 17354257 10.1002/dmrr.734

