# Supplementary material for: Machine learning–based skin nerve morphometry for diabetic neuropathy: diagnostic and clinical implications
Source: Brain Commun. 2026 Mar 31;8(2):fcag113. doi: 10.1093/braincomms/fcag113 (PMC13080513; doi:10.1093/braincomms/fcag113)
Supplement: fcag113_Supplementary_Data [file fcag113_supplementary_data.pdf]

Supplementary Table 1. Kernel used in the custom-convolution filtering.

0	0	-1	0	0
0	-1	-1	-1	0
-1	-1	12	-1	-1
0	-1	-1	-1	0
0	0	-1	0	0

Supplementary Table 2. Intrarater and interrater reliability analysis.

	Intrarater reliability		Interrater reliability	
	ICC (95% CI)	P value	ICC (95% CI)	P value
IENFa/A				
AA	0.978 (0.968–0.986)	-	0.979 (0.968–0.986)	-
CF	0.940 (0.917–0.956)	<0.001	0.953 (0.932–0.969)	<0.001
ED	0.954 (0.939–0.966)	<0.001	0.940 (0.912–0.958)	<0.001
IENFa/P				
AA	0.965 (0.955–0.974)	-	0.961 (0.933–0.979)	-
CF	0.923 (0.898–0.943)	<0.001	0.907 (0.882–0.937)	<0.001
ED	0.934 (0.914–0.954)	0.008	0.912 (0.882–0.937)	<0.001
Epidermis parameter				
Area	0.9997 (0.999-1.000)	-	0.917 (0.884-0.937)	-
Perimeter	0.998 (0.997-1.000)	-	0.958 (0.926-0.980)	-

ICC: intraclass correlation coefficient

[*]Cluster bootstrap method with pairwise comparison and Holm-Bonferroni correction

Supplementary Table 3. Linear regression analysis of different IENFas and IENFd to age and sex in the control group.

		Age	Sex
Univariate analysis			
IENFd		-0.101 (-0.153, -0.049)***	-1.215 (-2.743, 0.313)
IENFa/A	AA	-0.010 (-0.019, -0.001)*	-0.214 (-0.464, 0.037)
	CF	-0.009 (-0.018, 0.0004)	-0.271 (-0.516, -0.026)*
	ED	-0.024 (-0.047, -0.002)*	-0.622 (-1.225, -0.119)*
IENFa/P	AA	-0.005 (-0.008, -0.002)***	-0.020 (-0.099, 0.059)
	CF	-0.004 (-0.007, -0.001)**	-0.041 (-0.119, 0.037)
	ED	-0.011 (-0.017, -0.004)**	-0.091 (-0.278, 0.097)
Multivariate analysis without standardization			
IENFd		-0.105 (-0.156, -0.055)***	-1.453 (-2.814, -0.093)*
IENFa/A	AA	-0.011 (-0.020, -0.002)**	-0.238 (-0.480, 0.004)
	CF	-0.010 (-0.019, -0.001)*	-0.293 (-0.531, -0.054)*
	ED	-0.027 (-0.048, -0.005)*	-0.681 (-1.263, -0.099)*
IENFa/P	AA	-0.005 (-0.008, -0.002)***	-0.031 (-0.104, 0.042)
	CF	-0.004 (-0.007, -0.002)**	-0.050 (-0.123, 0.023)
	ED	-0.011 (-0.018, -0.005)**	-0.115 (-0.289, 0.058)
Multivariate analysis with IENF and age standardization			
IENFd		-0.464 (-0.688, -0.241)***	-0.494 (-0.957, 0.032)*
IENFa/A	AA	-0.290 (-0.531, -0.048)*	-0.489 (-0.987, 0.009)
	CF	-0.262 (-0.502, -0.022)*	-0.606 (-1.100, -0.111)*
	ED	-0.291 (-0.53, -0.052)*	-0.576 (-1.068, -0.083)*
IENFa/P	AA	-0.421 (-0.655, -0.186)***	-0.206 (-0.69, 0.277)
	CF	-0.373 (-0.611, -0.135)**	-0.335 (-0.826, 0.155)
	ED	-0.402 (-0.638, -0.167)**	-0.322 (-0.807, 0.162)

AA: automated annotation; CF: custom-convolution filtering; ED: edge detection

*<0.05, **<0.01, ***<0.001

Supplementary Table 4. Diagnostic performance of area-based intraepidermal nerve fiber (IENFa) quantification based on IENF density-defined small-fiber neuropathy.

		Age	Sen. (%)	Spe. (%)	Youden's index	Threshold	AUC (P value)
AA	IENFa/A	<60	100	87.0	0.870	0.470	0.959*
		≧60	100	73.7	0.737	0.318	0.932*
		All	94.2	86.8	0.810	0.420	0.944*
	IENFa/P	<60	100	82.6	0.826	0.136	0.955 (0.464)
		≧60	97.3	84.2	0.815	0.105	0.945 (0.552)
		All	98.6	81.6	0.802	0.106	0.948 (0.756)
CF	IENFa/A	<60	81.3	95.7	0.770	0.700	0.940 (0.400)
		≧60	86.5	84.2	0.707	0.385	0.913 (0.409)
		All	91.3	78.9	0.702	0.385	0.918 (0.176)
	IENFa/P	<60	84.4	91.3	0.757	0.184	0.932 (0.117)
		≧60	94.6	84.2	0.788	0.094	0.935 (0.862)
		All	95.7	78.9	0.746	0.096	0.925 (0.290)
ED	IENFa/A	<60	90.6	91.3	0.819	1.220	0.951 (0.517)
		≧60	81.1	89.5	0.706	0.937	0.927 (0.856)
		All	82.6	92.1	0.747	1.194	0.932 (0.509)
	IENFa/P	<60	96.9	87.0	0.839	0.270	0.939 (0.247)
		≧60	97.2	84.2	0.814	0.216	0.940 (0.763)
		All	98.6	84.2	0.828	0.210	0.932 (0.611)

*Compared to IENFa/A via the Delong's test

A: area of the epidermis; P: perimeter of the epidermis

AA: automated annotation; CF: custom-convolution filtering; ED: edge detection

Graphic demonstration of the IENF segmentation

Introduction

All three approaches aim to quantify intraepidermal nerve fibers (IENFs), they differ in their underlying principles and levels of human supervision:

1. Automated annotation (machine learning–assisted segmentation)

This approach uses supervised pixel-level classification implemented via the Trainable Weka Segmentation plugin in FIJI. Manually labeled pixels representing IENFs and background are used to train a classifier, which then assigns each pixel to the corresponding class. This method measures the total intraepidermal nerve fiber area by directly segmenting nerve fibers based on learned image features (e.g., intensity and texture). Importantly, this approach is designed for accurate within-image segmentation under standardized staining and imaging conditions, rather than for building a globally generalizable classifier.

2. Edge detection–based method

This conventional image-processing approach relies on intensity gradients to identify fiber boundaries. After edge detection and binarization, morphological operations are applied before particle analysis within the epidermal region of interest. This method primarily captures nerve fiber contours and estimates nerve fiber area based on detected edges, without any machine learning or training process.

3. Custom convolution filtering–based method

This approach applies a predefined convolution kernel to enhance linear, fiber-like structures in the image. Following thresholding and morphological processing, particle analysis is performed to quantify nerve fiber area. Unlike the Weka-based approach, this method does not adapt to data through training; instead, it measures nerve fiber signals emphasized by the predefined filter.

Split the channel to get green channel for IENF segmentation

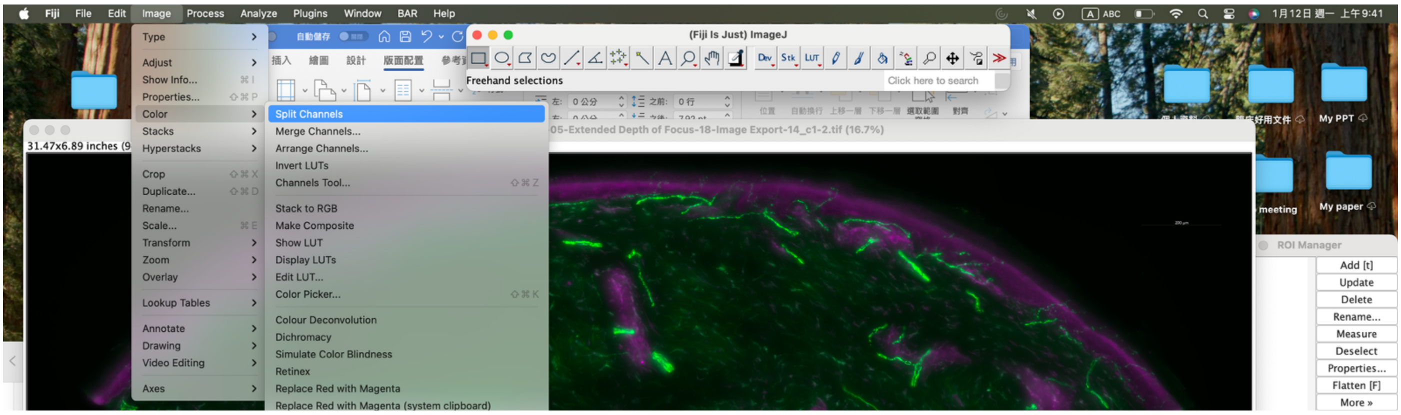

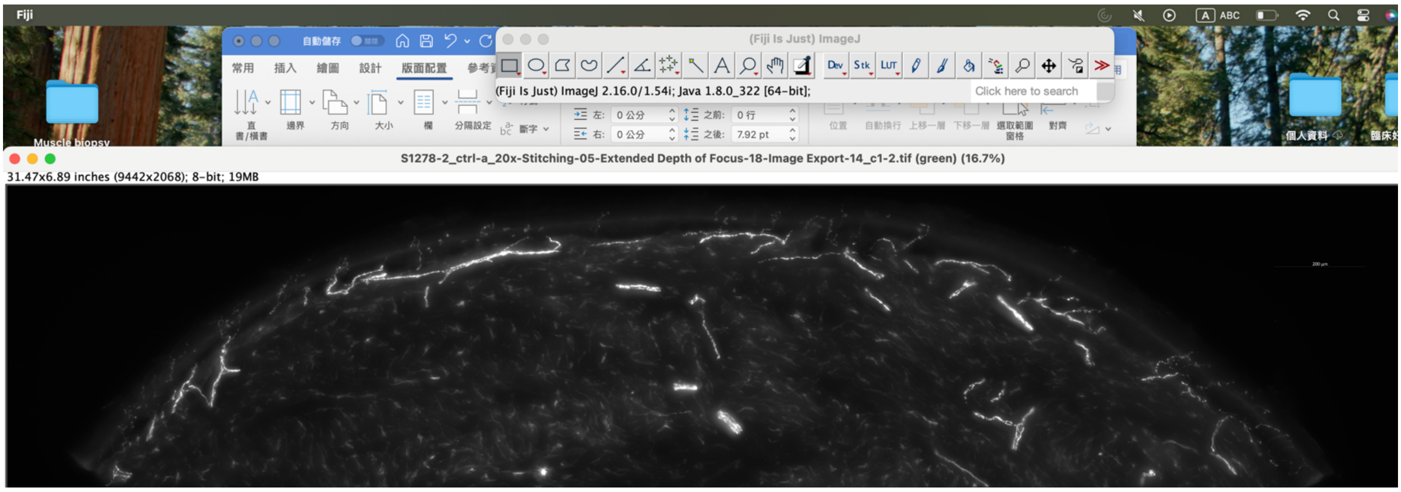

Automatic annotation:

Weka-based automated annotation (AA) was implemented using the Trainable Weka Segmentation plugin in FIJI. Given the consistent staining protocol and image acquisition settings across samples, a prototype classifier was initially trained using two single representative skin biopy images from one subject. Supervised annotations were manually provided to label intraepidermal nerve fibers and background regions.

This prototype classifier was subsequently applied to all images to generate an initial segmentation. For each skin biopsy section, minor case-specific refinements were performed by additional supervised annotations using the same Weka framework, followed by retraining to optimize segmentation quality. No large-scale dataset splitting was performed, as the objective of this approach was pixel-level segmentation within a standardized imaging pipeline rather than the development of a globally generalizable classifier.

-Training a prototype classifier

- Set scales

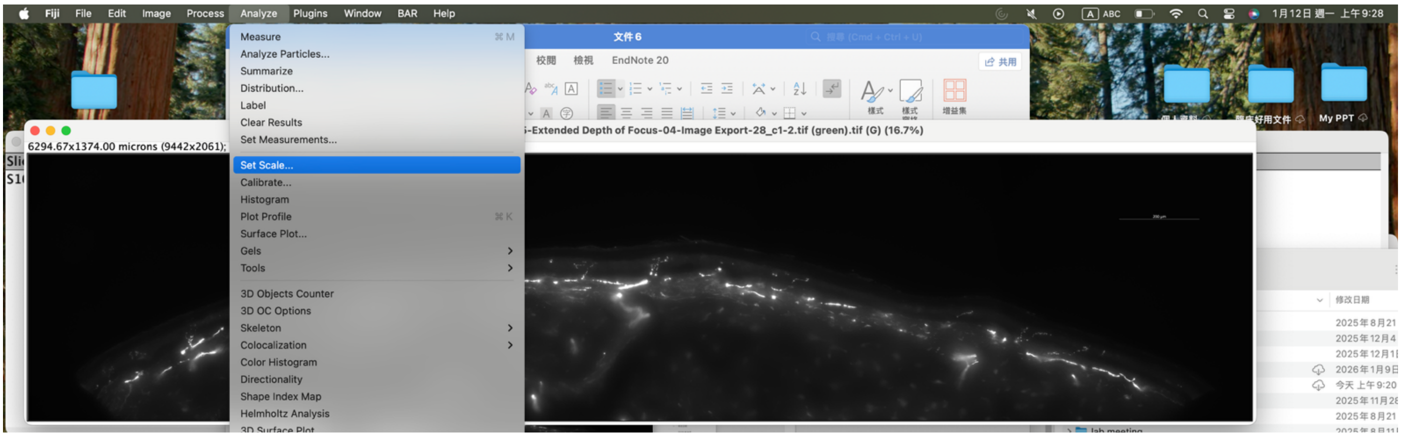

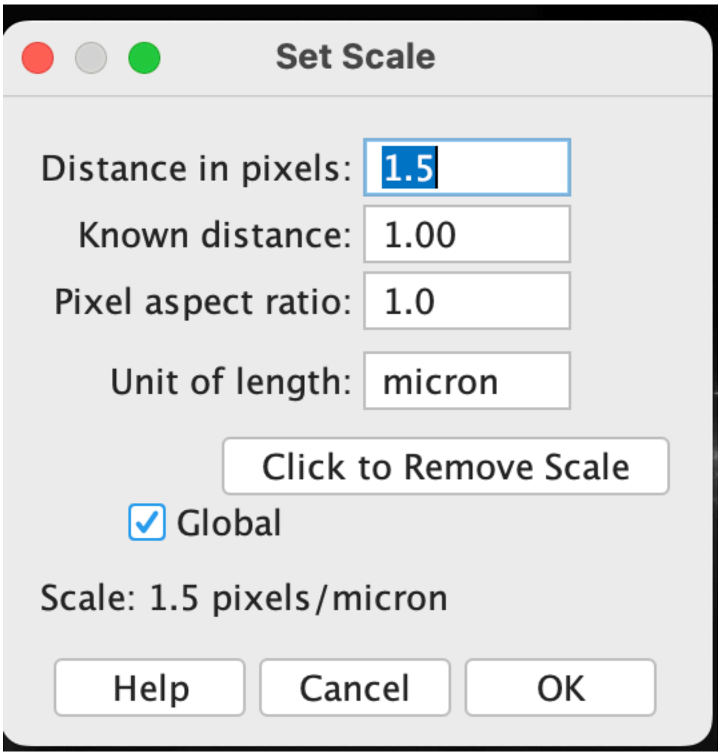

- Set up the memory and thread

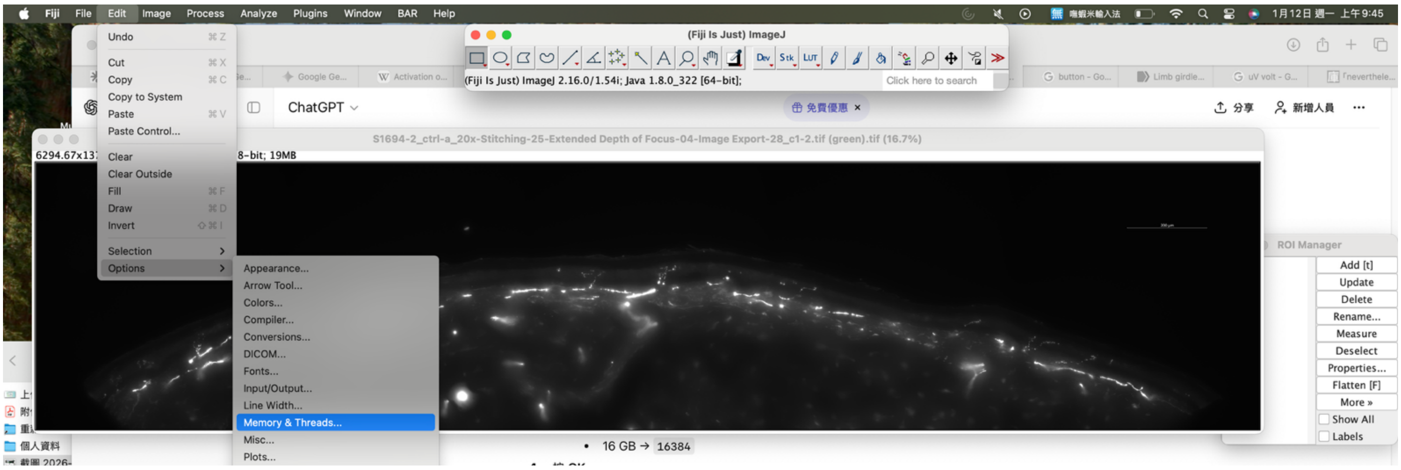

- Train a protype classifier

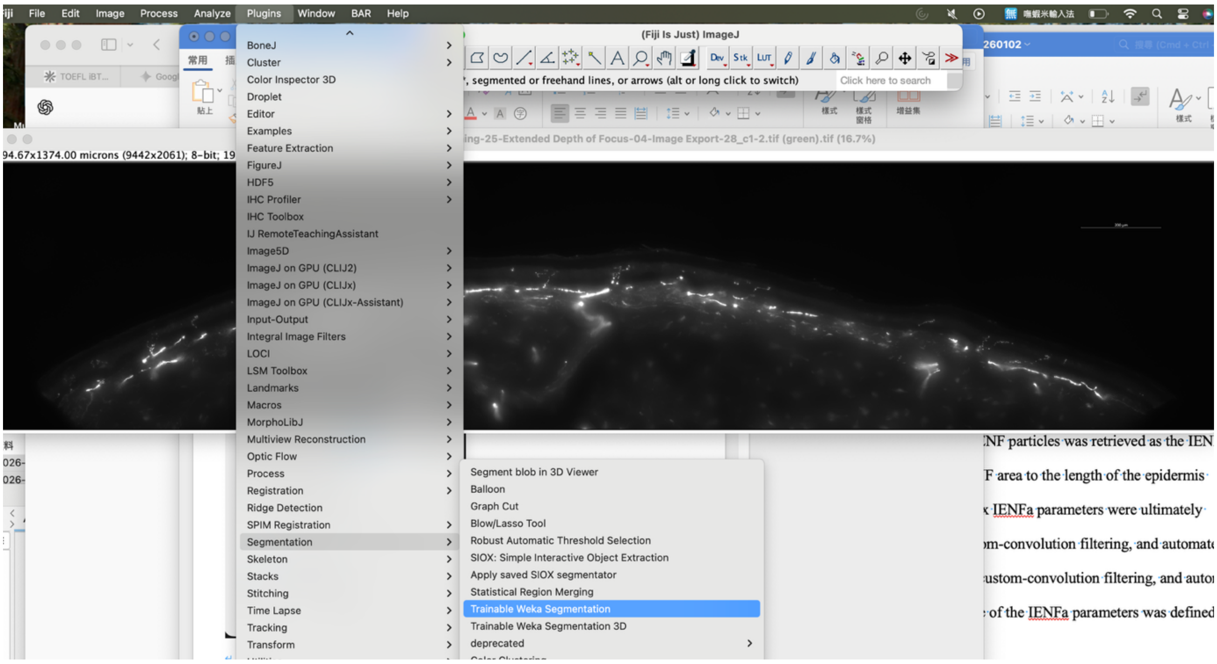

Label the pixel manually to class 1 or 2, then start training.

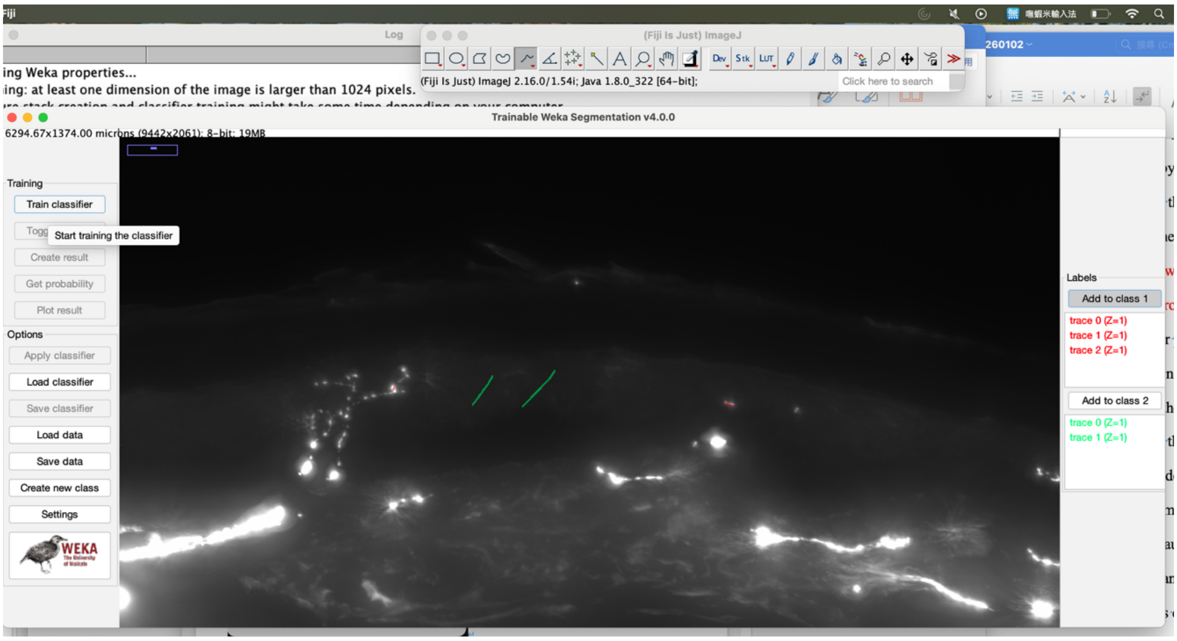

Check the first classification, you could take off “Toggle overlay” to check more precisely.

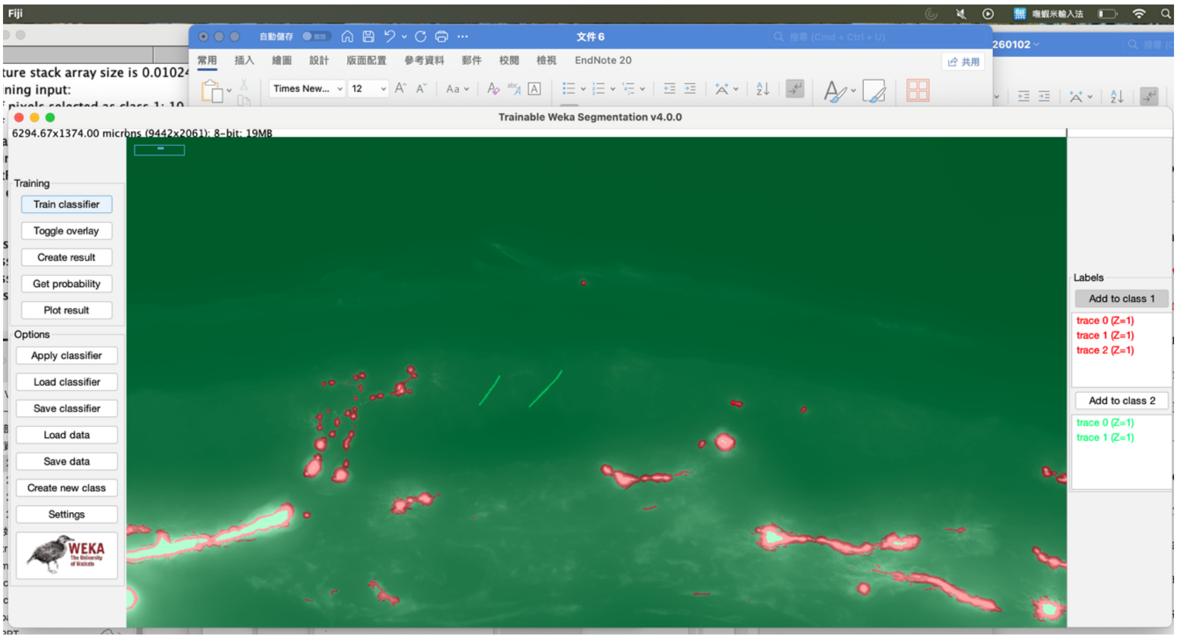

If not satisfied, label the pixel that being classified like your though, then repeat training (push "Train classifier) until you satisfy.

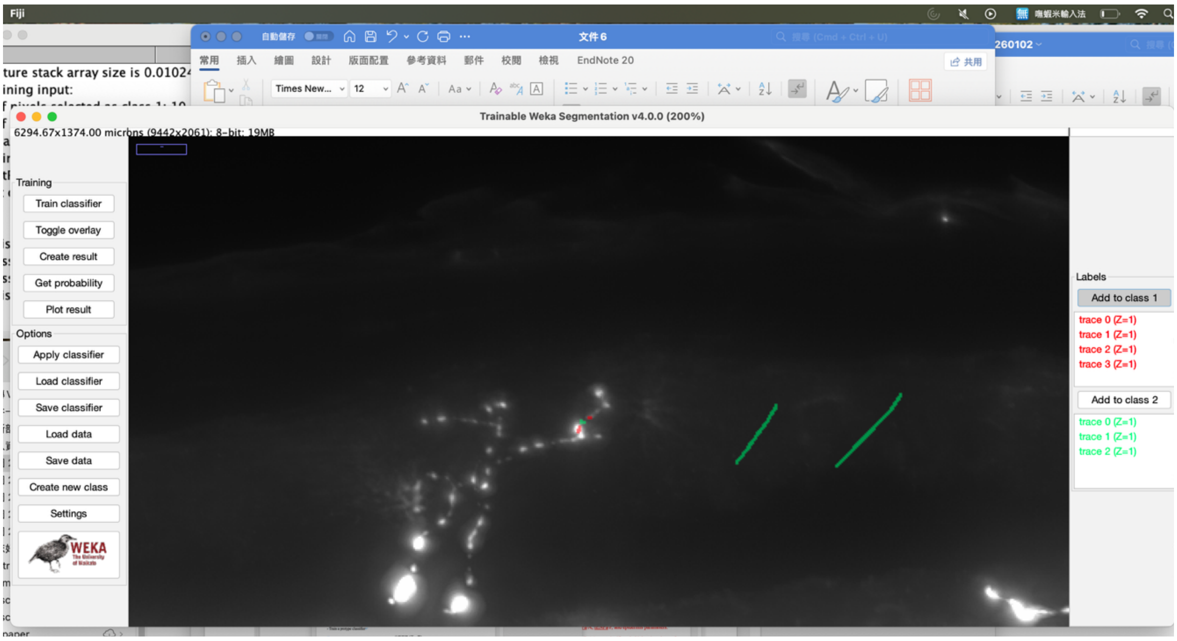

Save the trained protype classified.

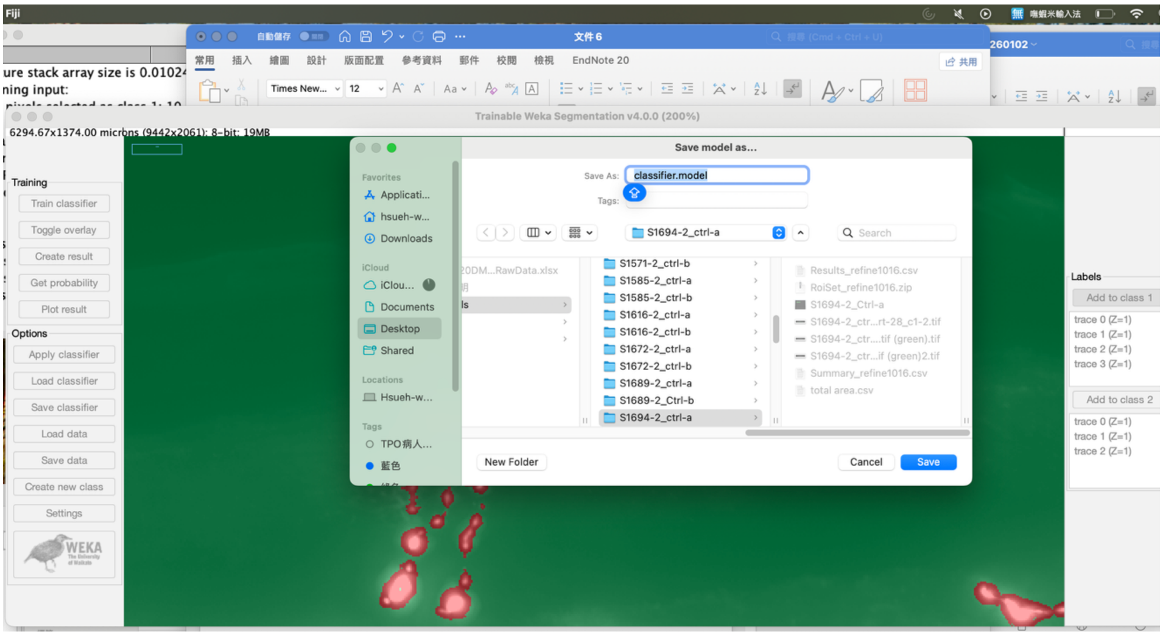

- Segment of the IENF

Open the image you want to classify, then open the Weka trainable segmentation plug-in. Load the classifier

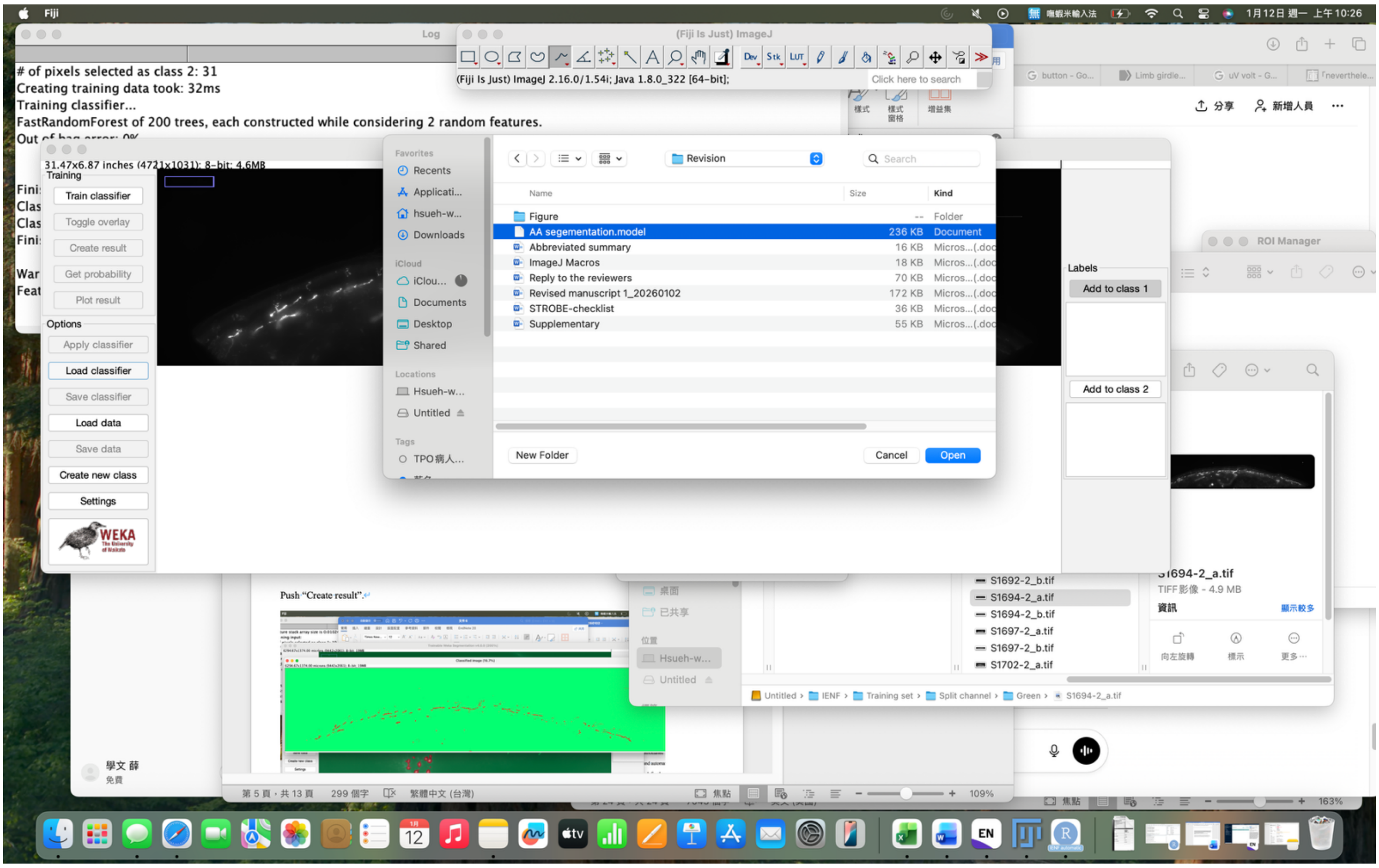

Push “Create result”.

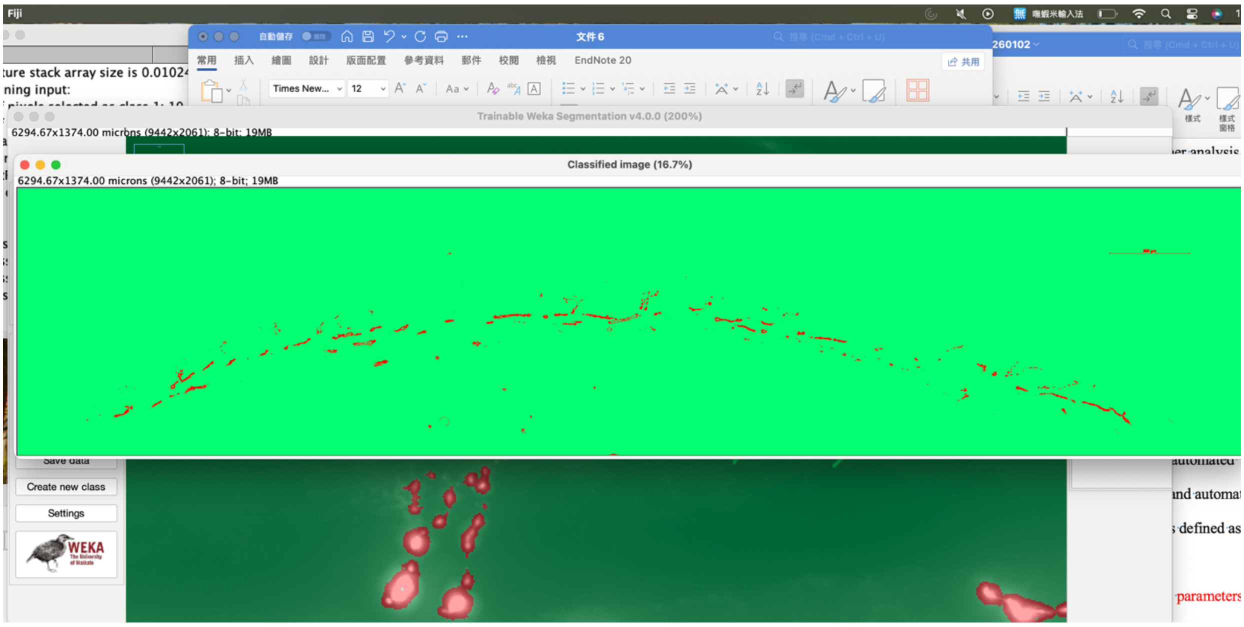

You could check the result. If not satisfied, then label the pixel you do not agree into Class 1 or Class 2. Then push "Train classifier" again. Then repeat the course until you satisfy the result.

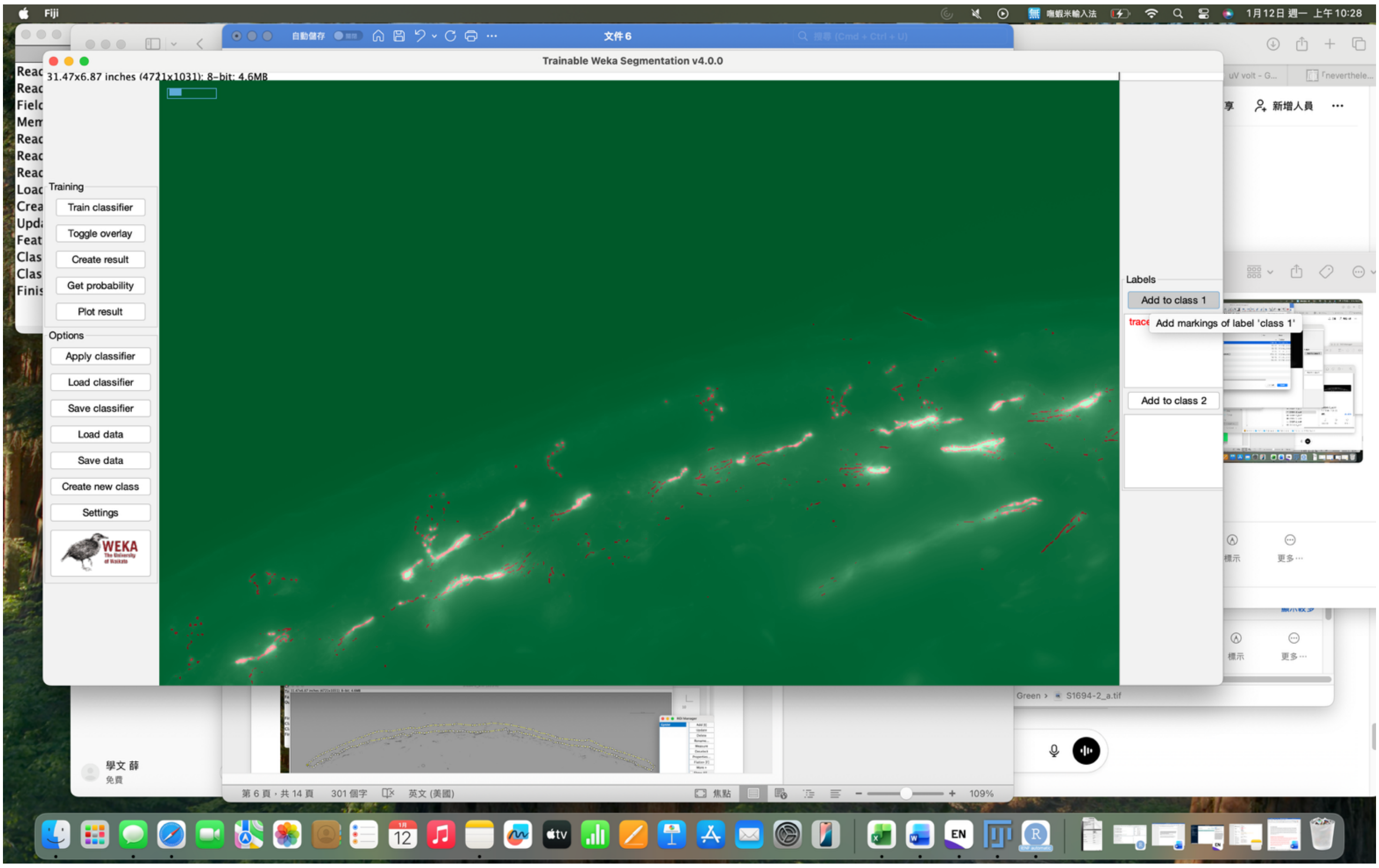

After you satisfy the segmentation, then push "Create result".

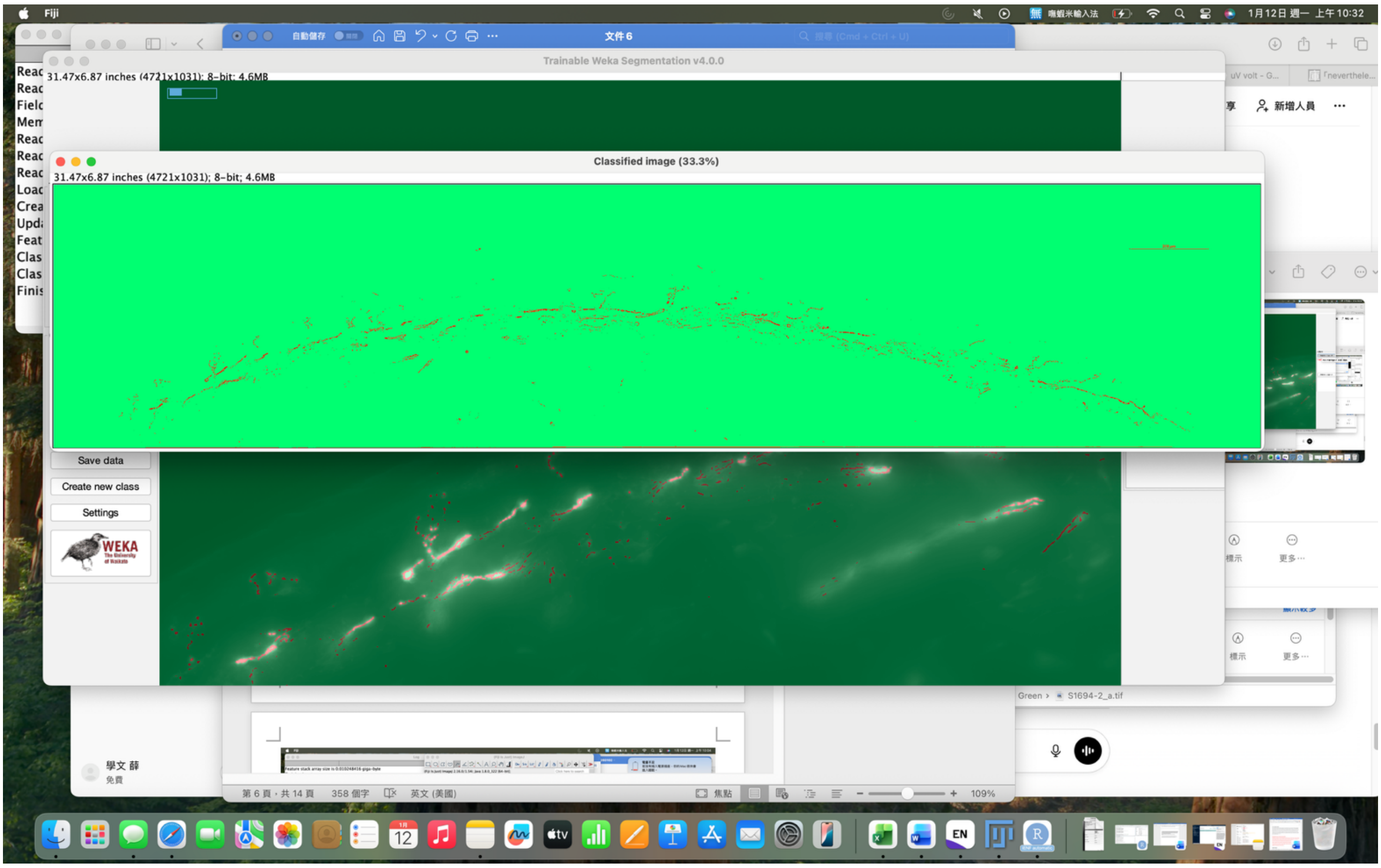

Then change to 8-bit, open the ROI manager, and load the epidermis segmentation ROI

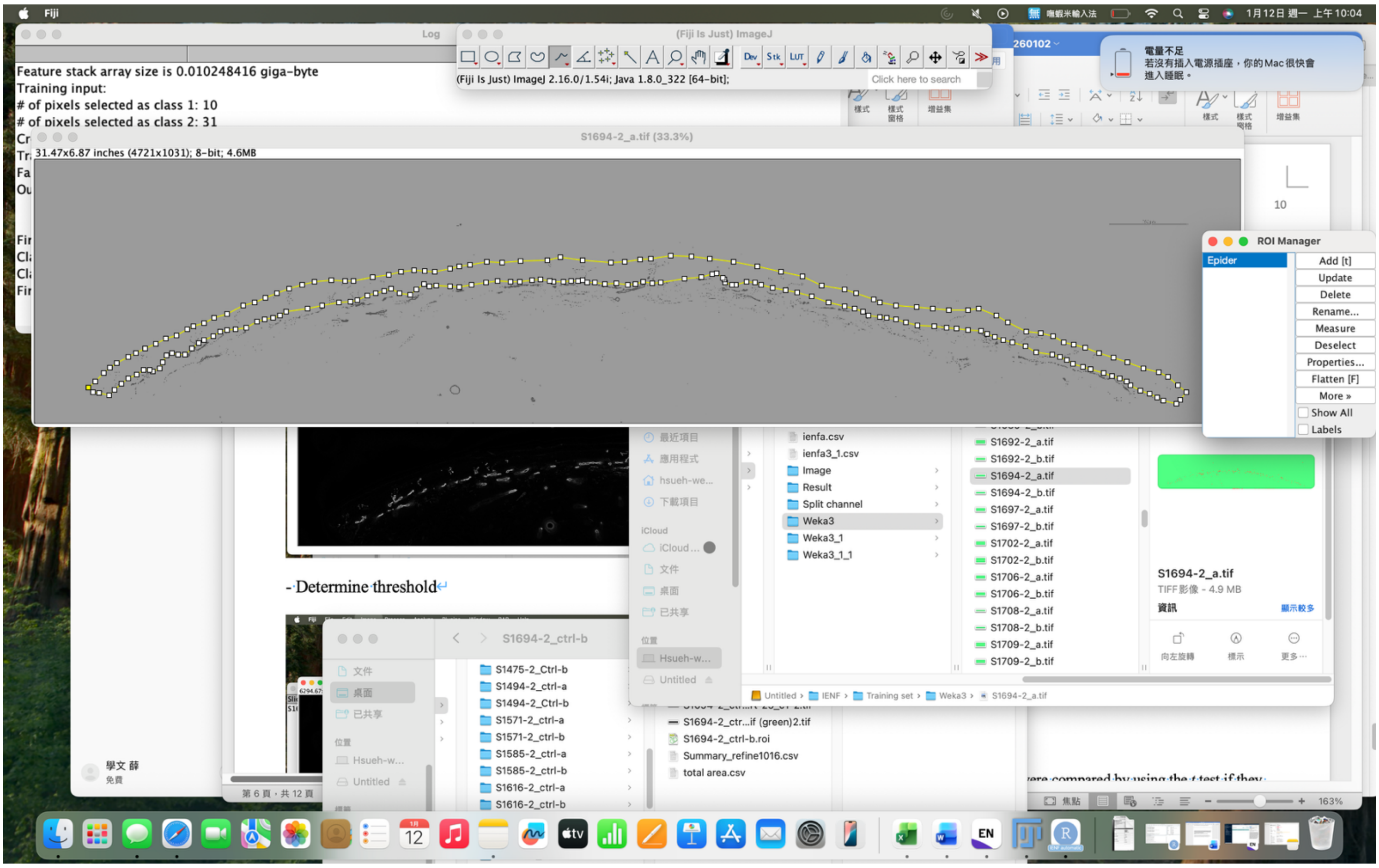

Adjust the threshold and analyze the particles

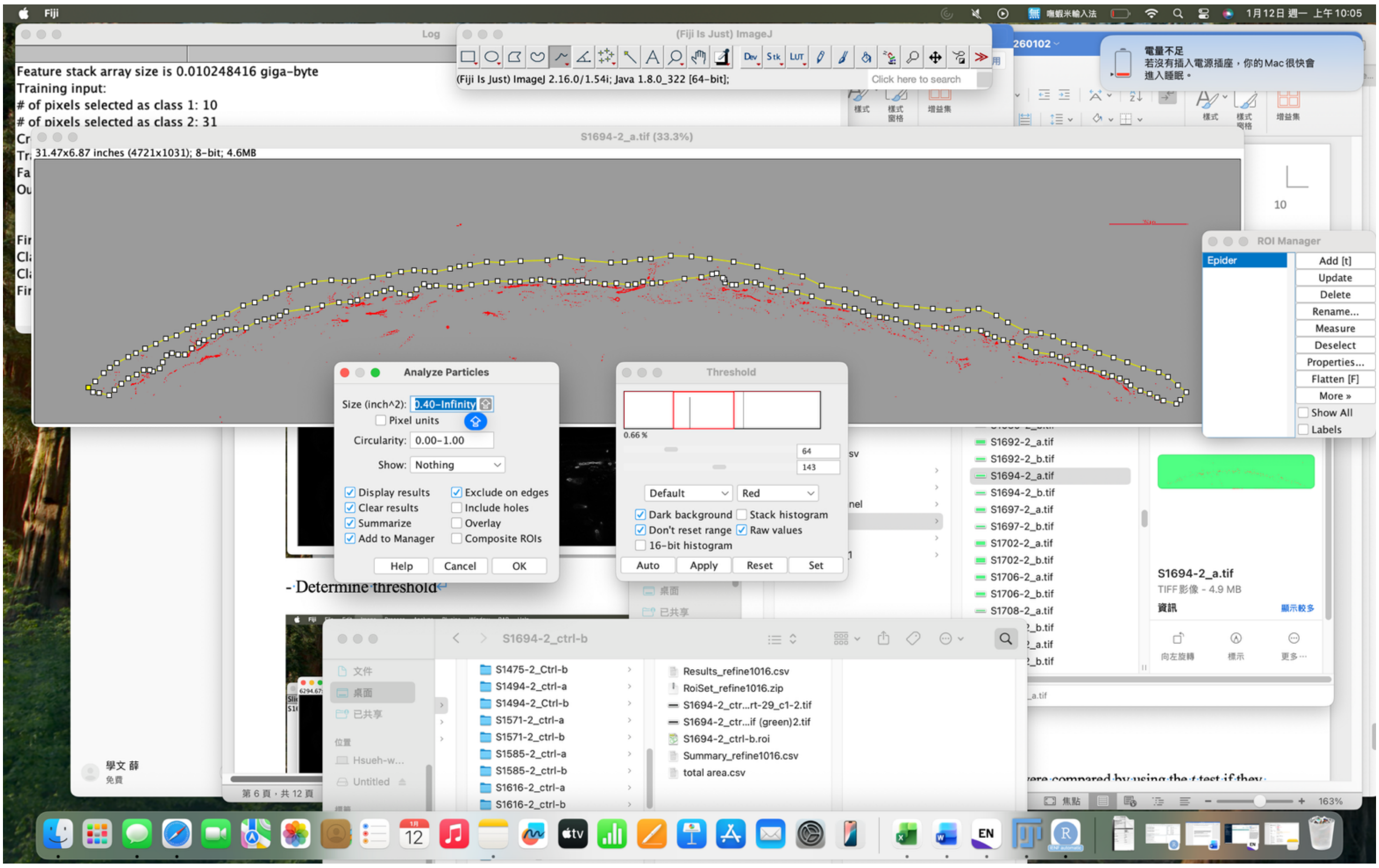

Set the details, and push “OK”

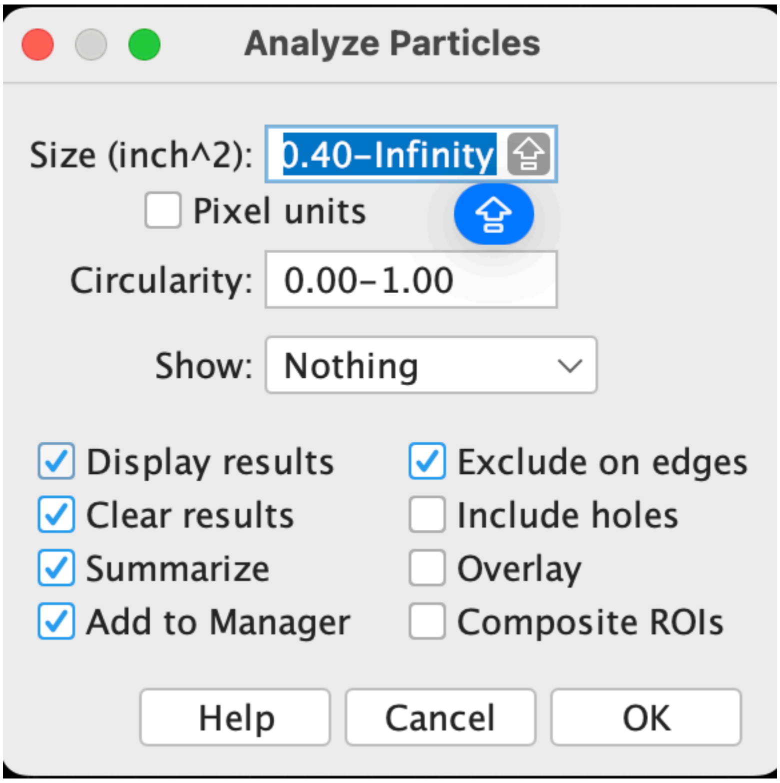

- Get the results (Total Area)

Summary

Slice	Count	Total Area	Average Size	%Area	Mean	Perim.	Circ.	Solidity
S1694-2_a.tif	318	922.222	2.900	0.628	85.000	5.515	0.968	0.907

Edge detection

- Set scales

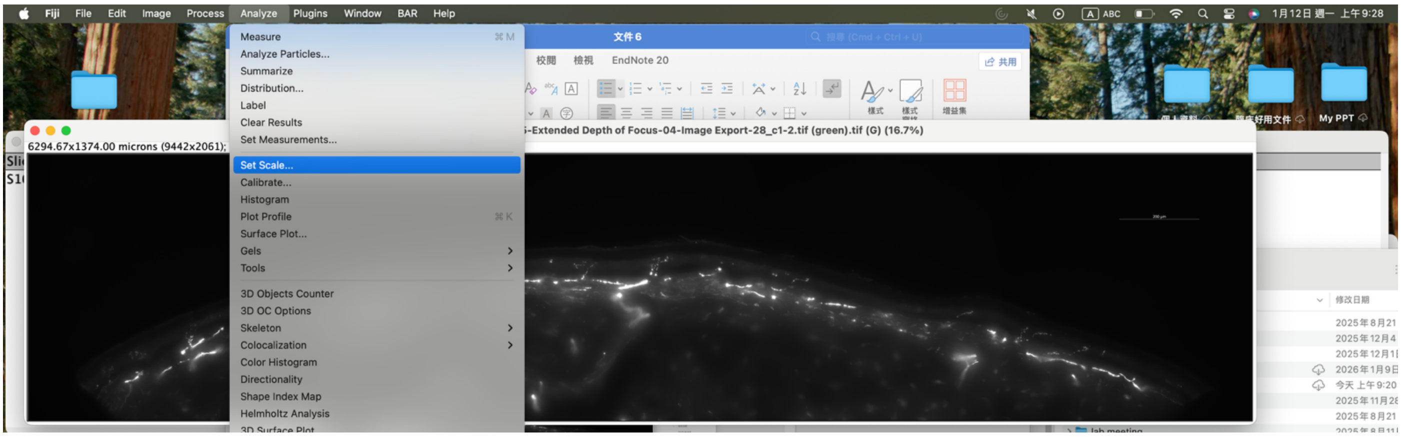

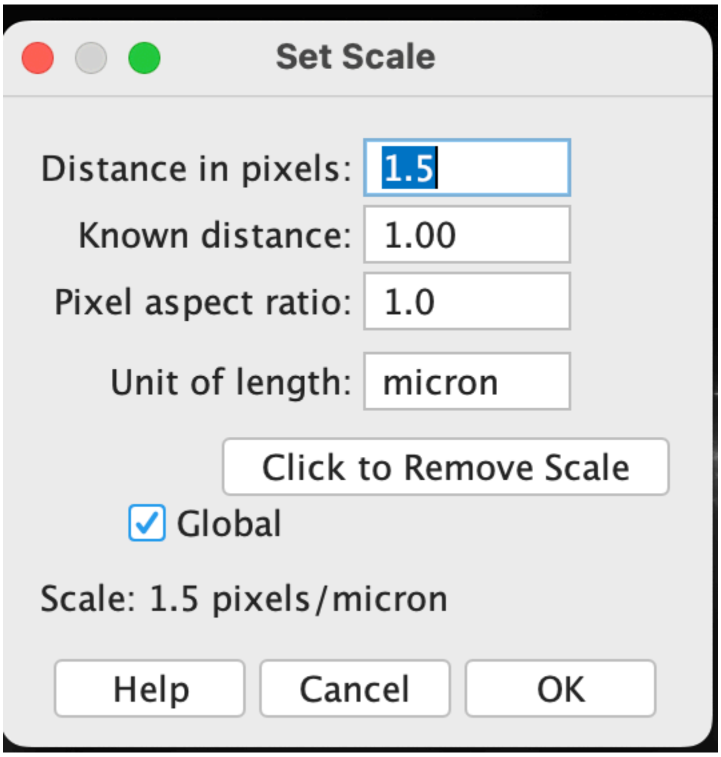

- Find edges

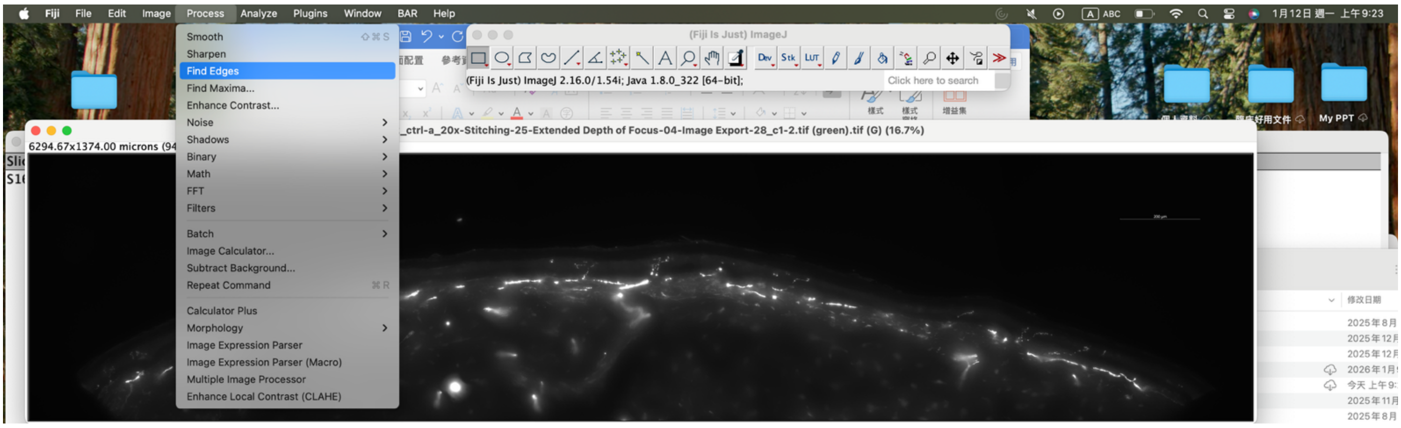

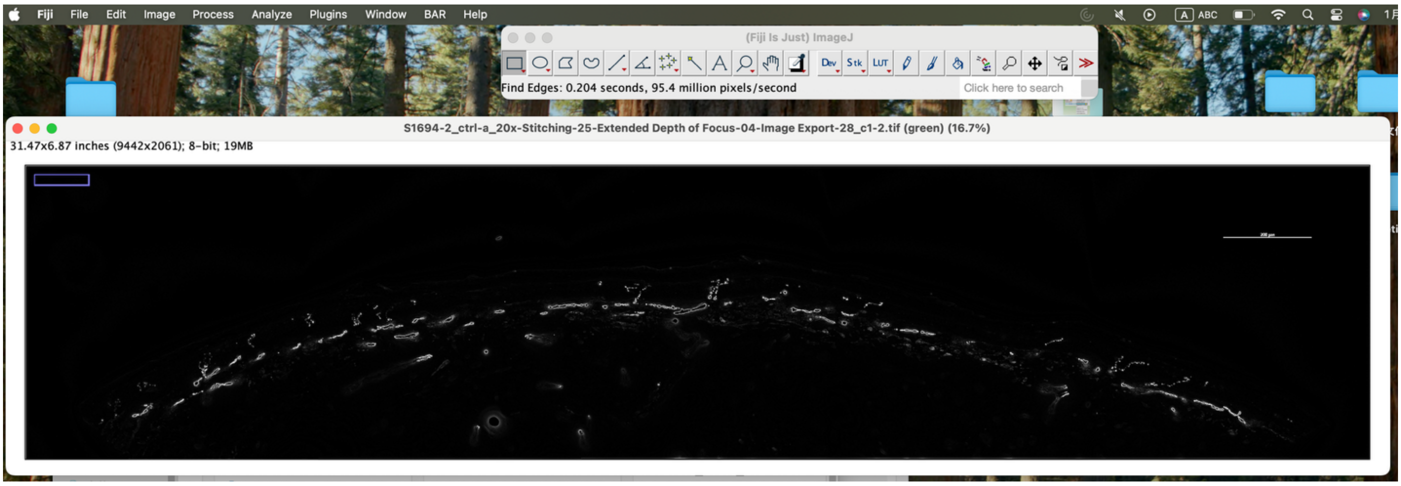

- Determine threshold, and push “Apply” to get binary images.

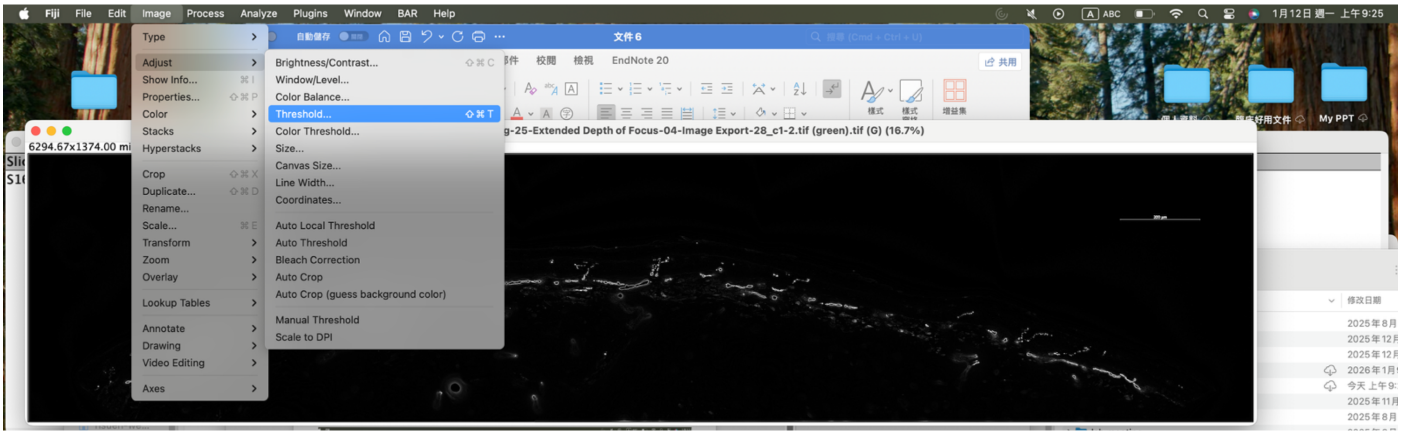

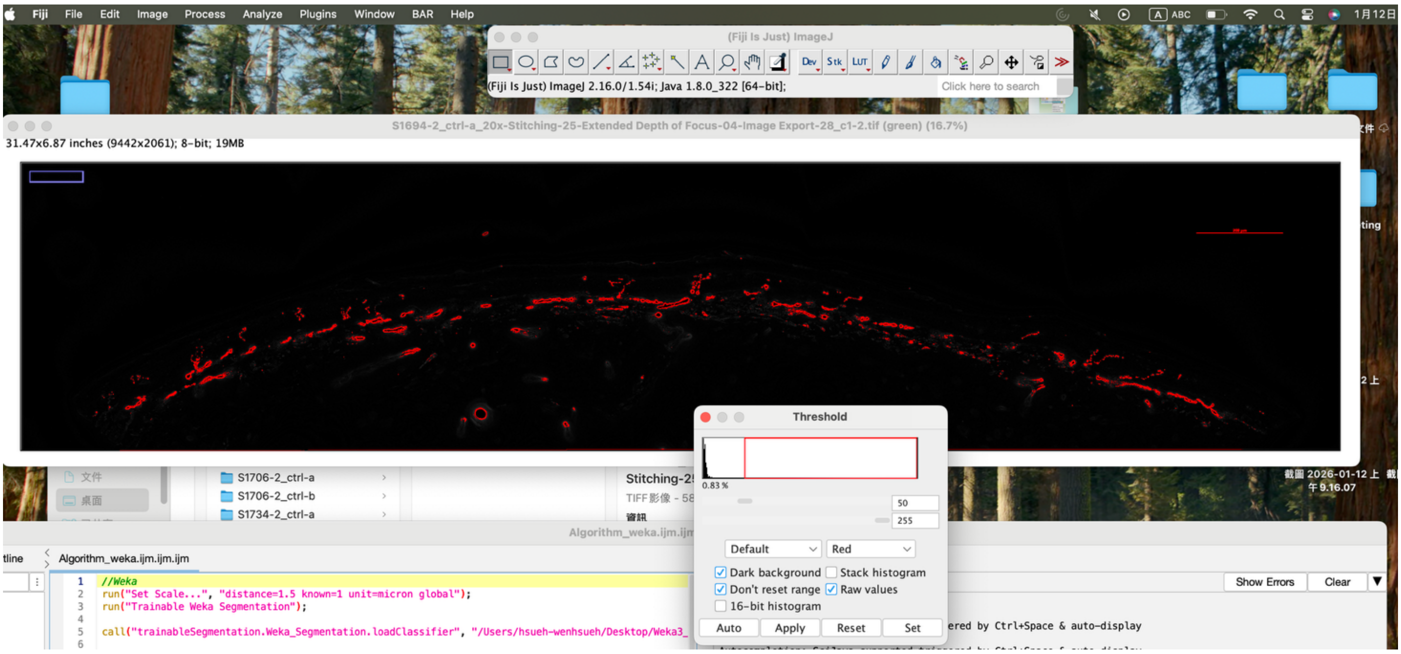

“Close-”

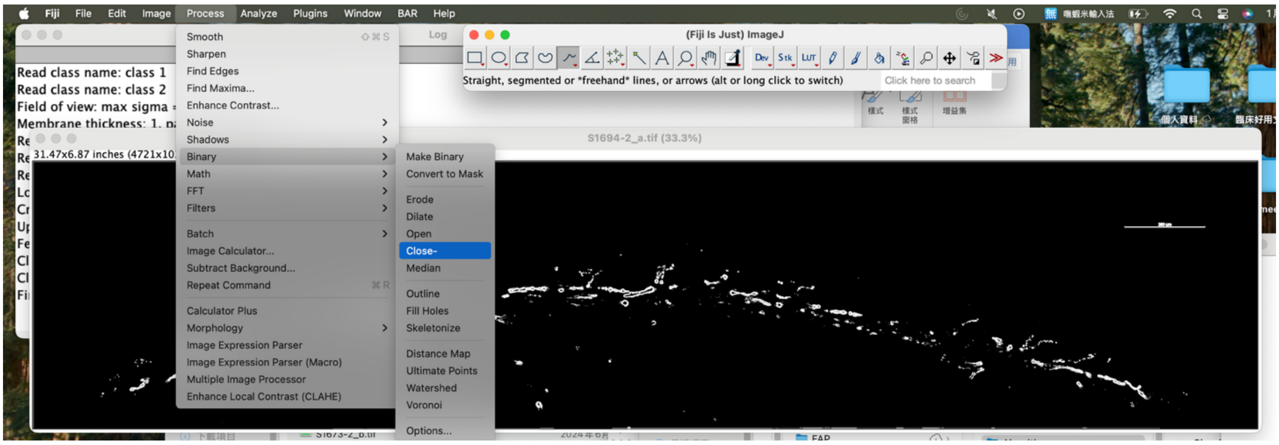

“Fill Holes”

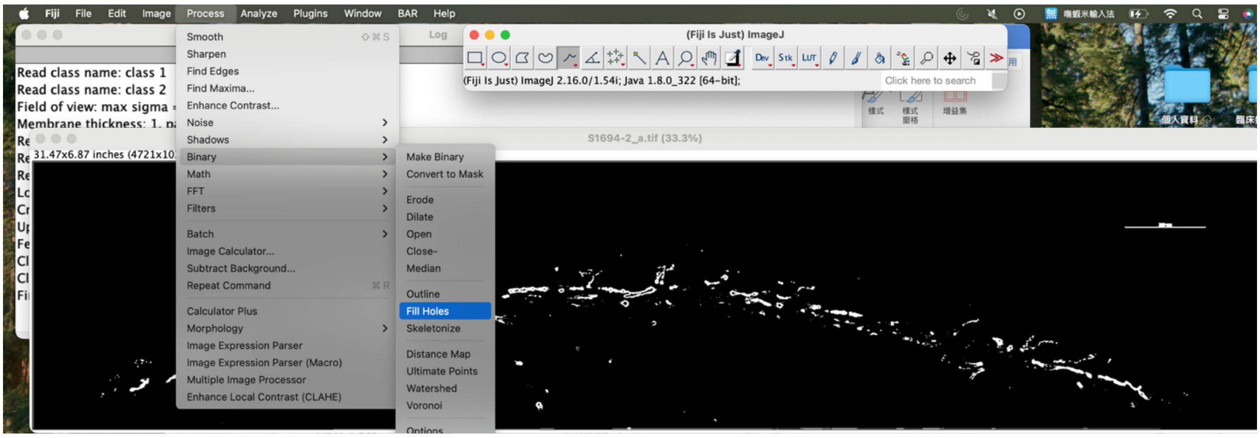

Then open the ROI manager, and load the epidermis segmentation ROI

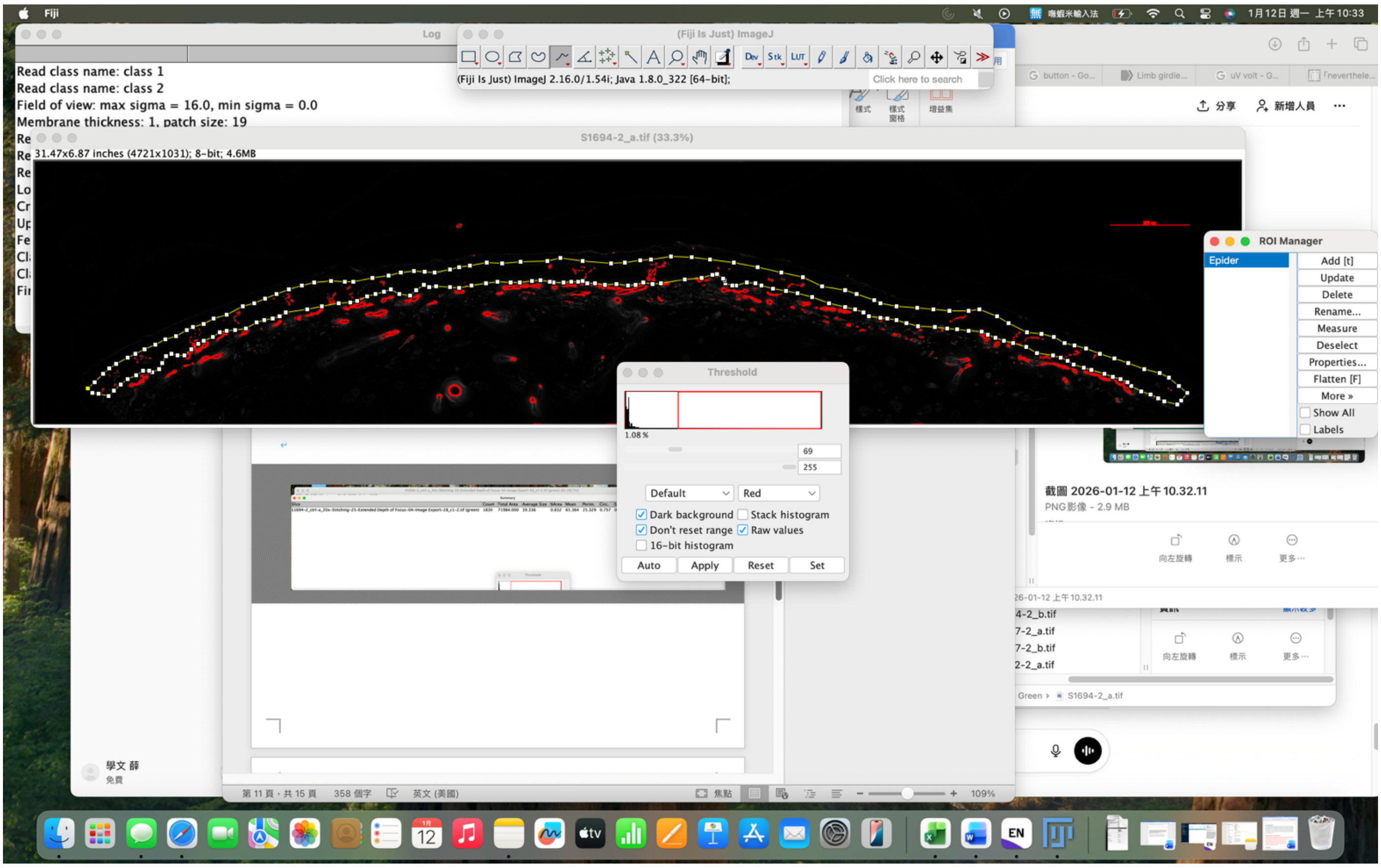

- Analyze particles

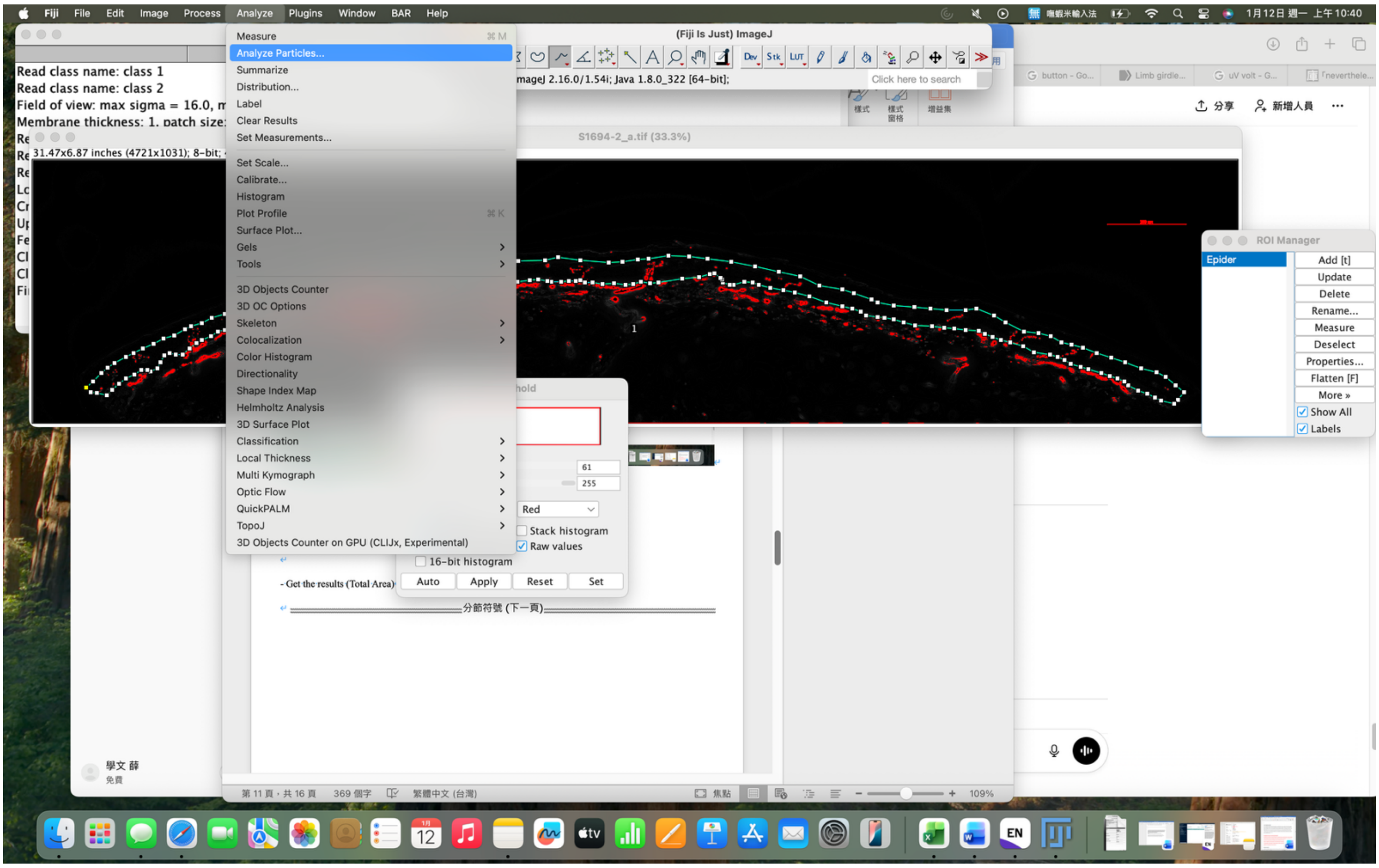

Set the details, and push “OK”

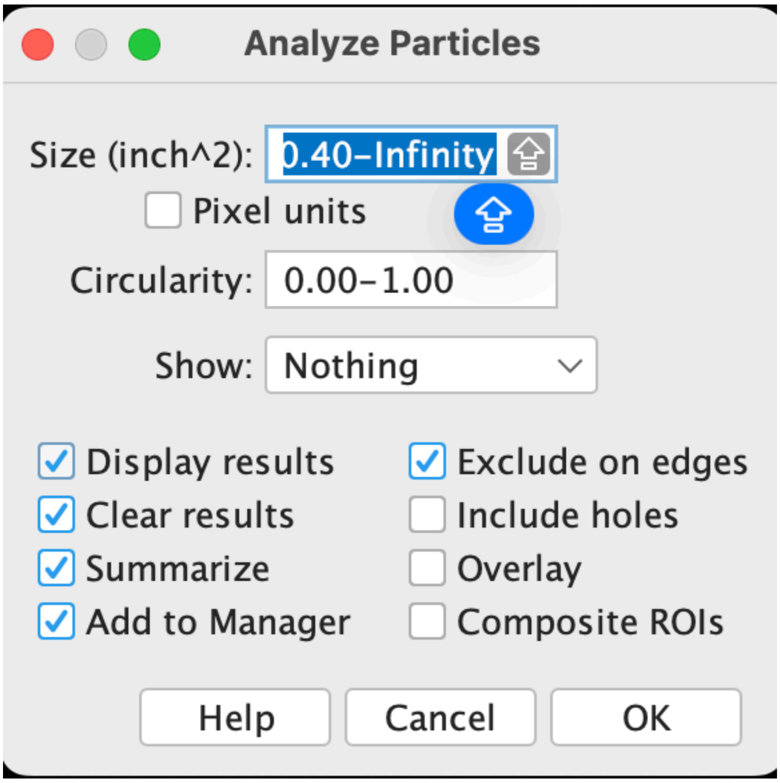

- Get the results (Total Area)

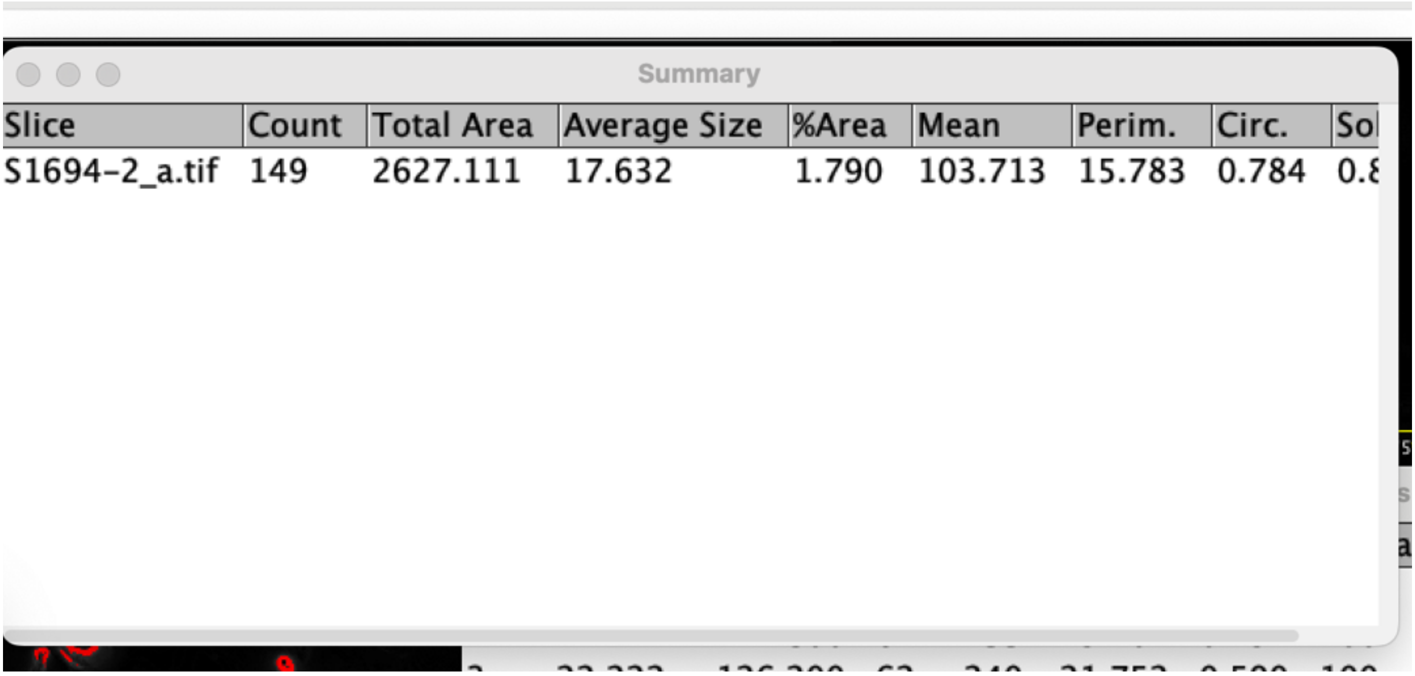

Summary

Slice	Count	Total Area	Average Size	%Area	Mean	Perim.	Circ.	So
S1694-2_a.tif	149	2627.111	17.632	1.790	103.713	15.783	0.784	0.8

Custom-convolution filtering

- Set scales

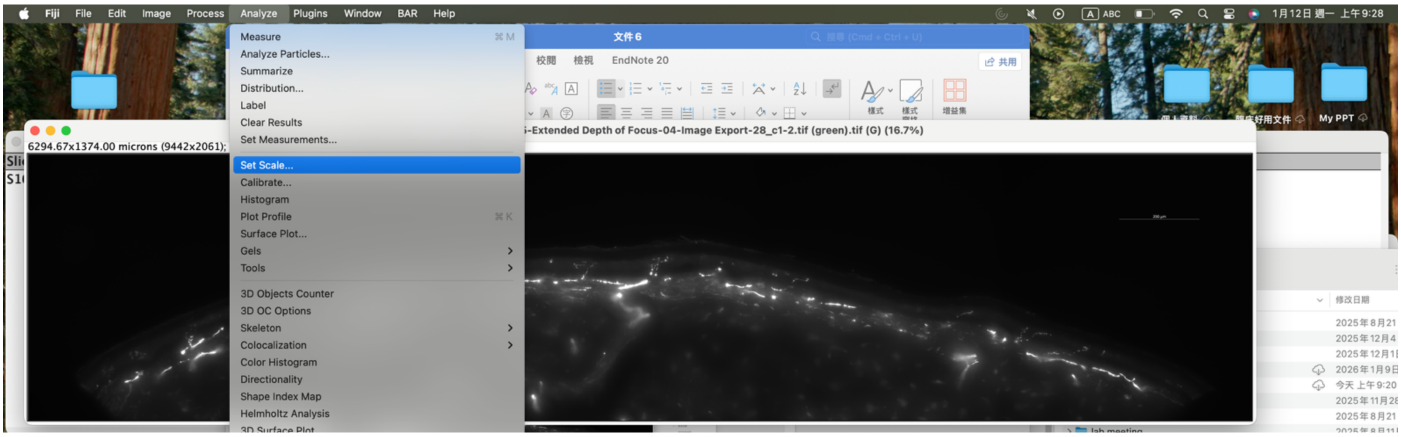

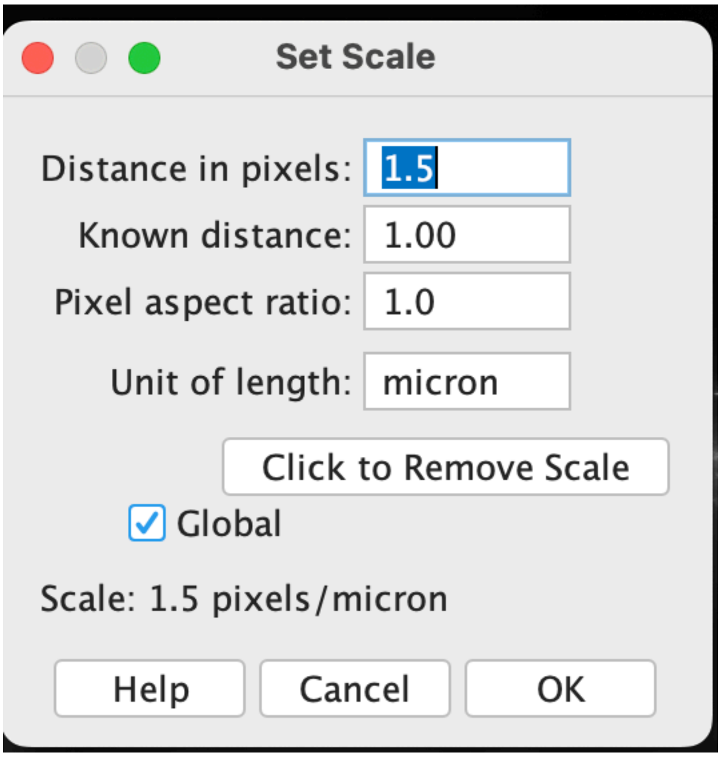

- Set up the Kernel

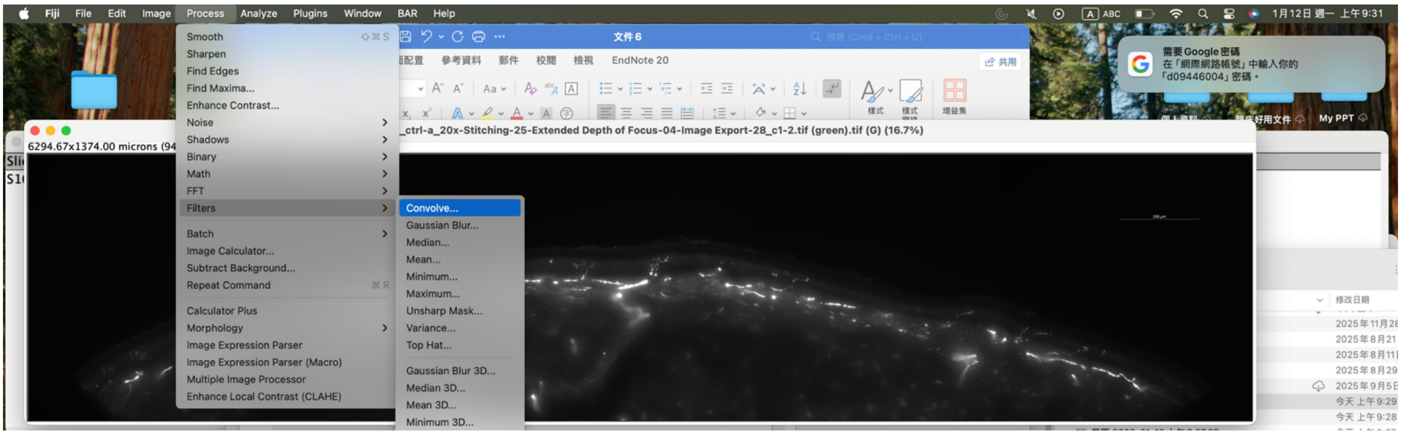

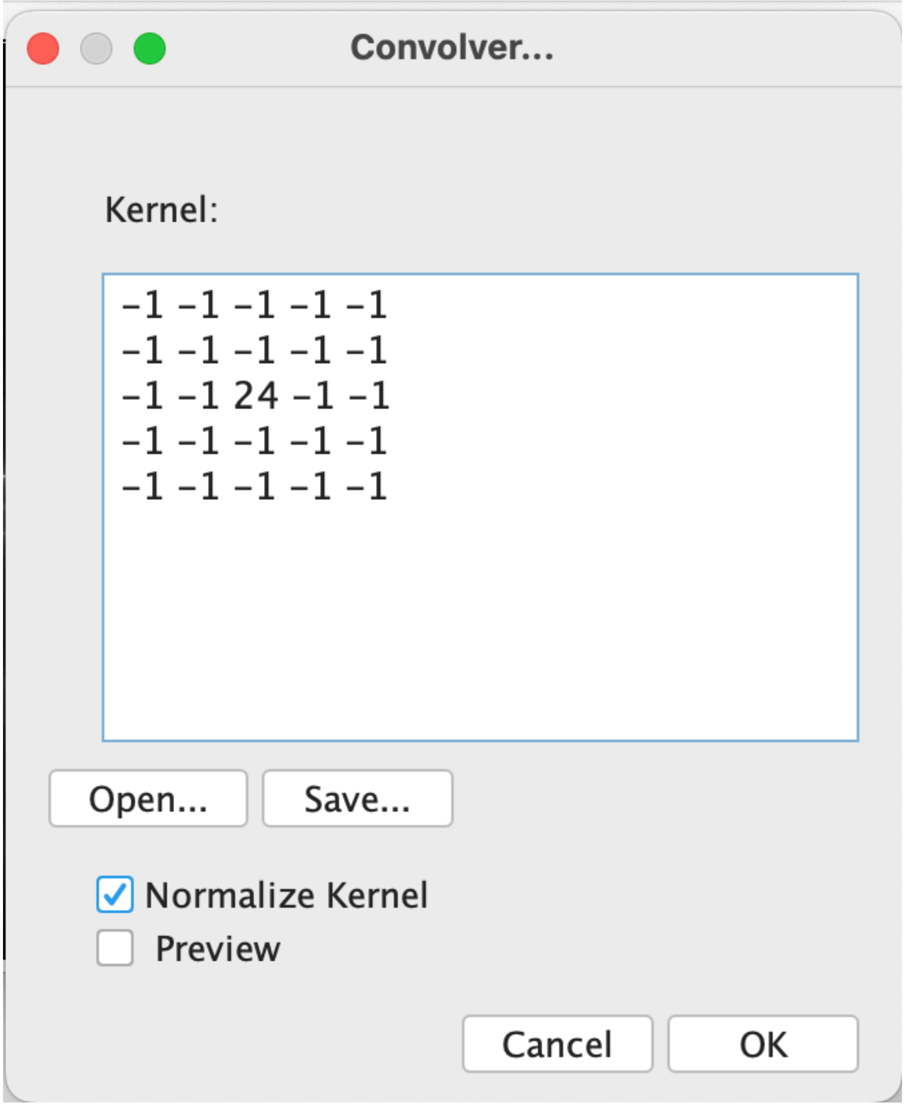

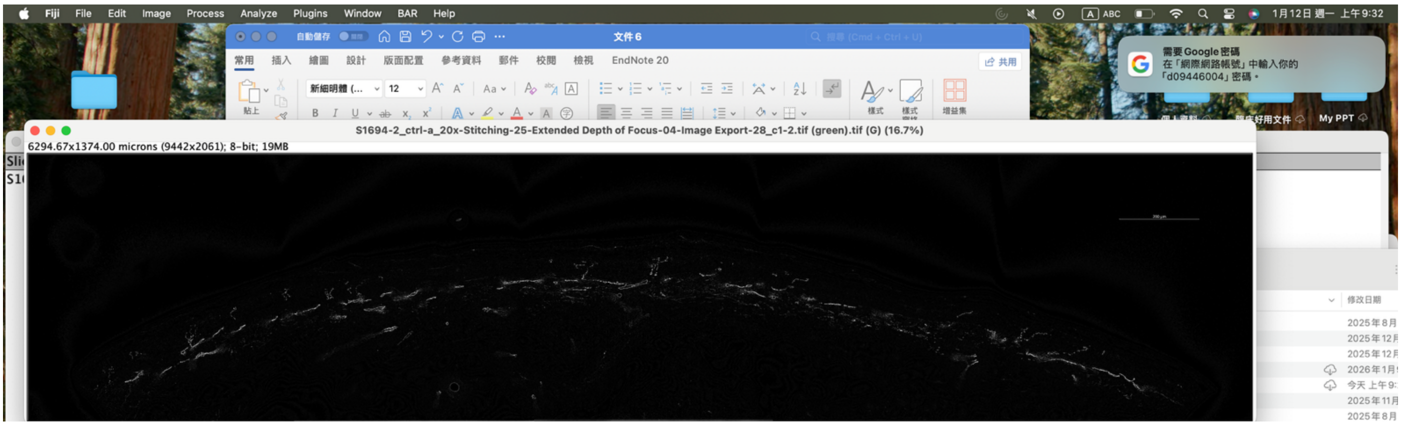

- Determine threshold, and push “Apply” to get binary images.

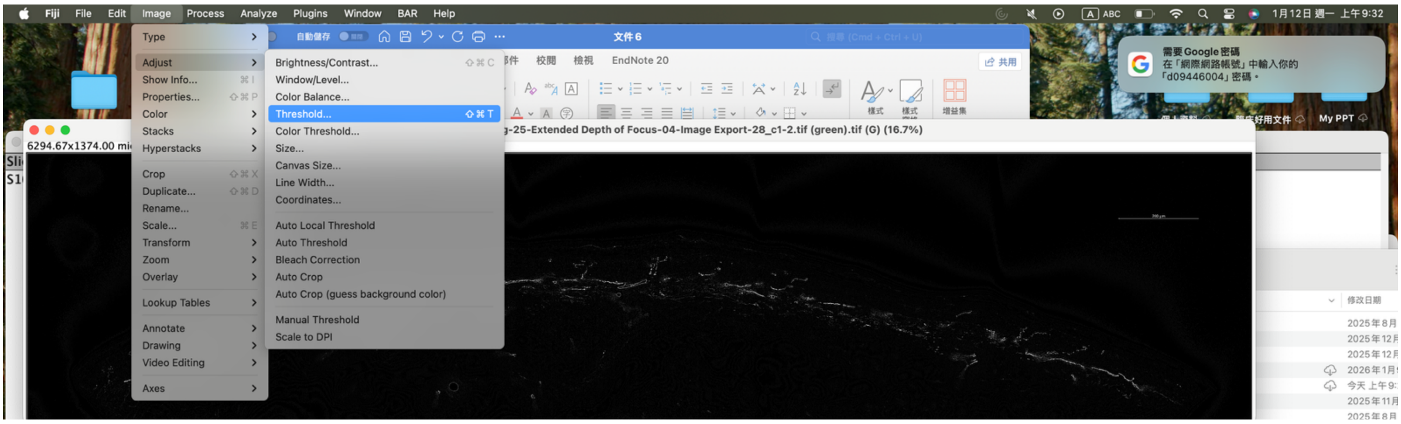

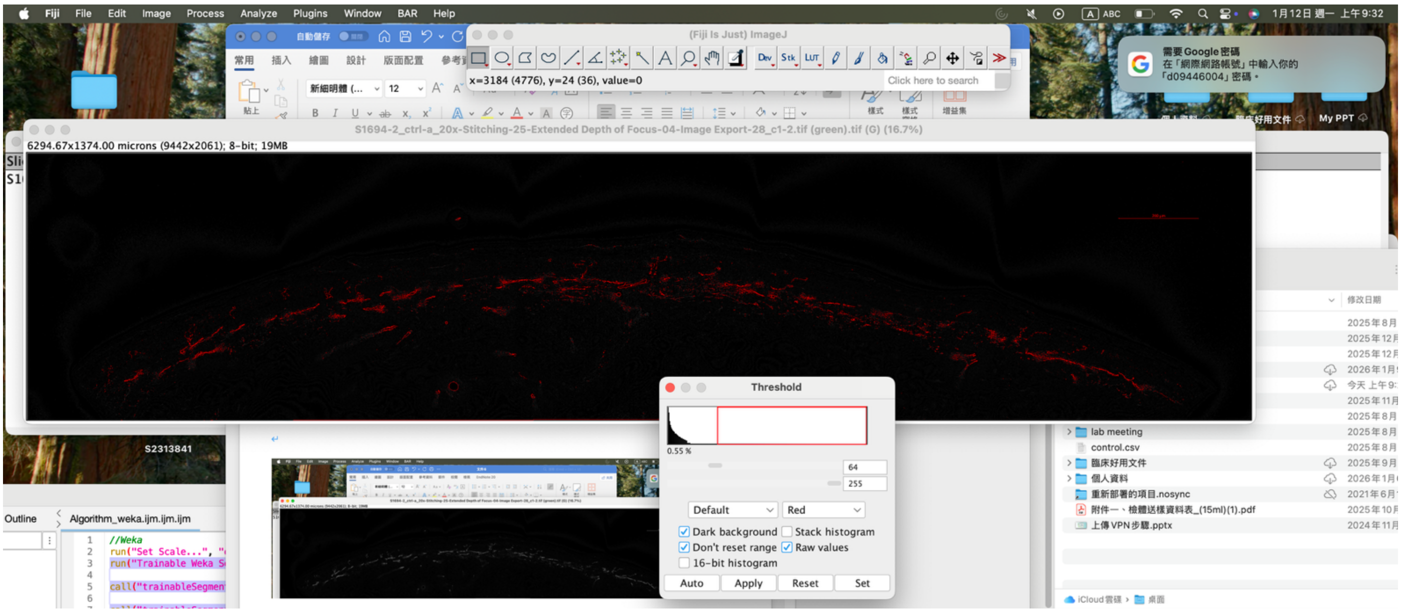

“Close-”

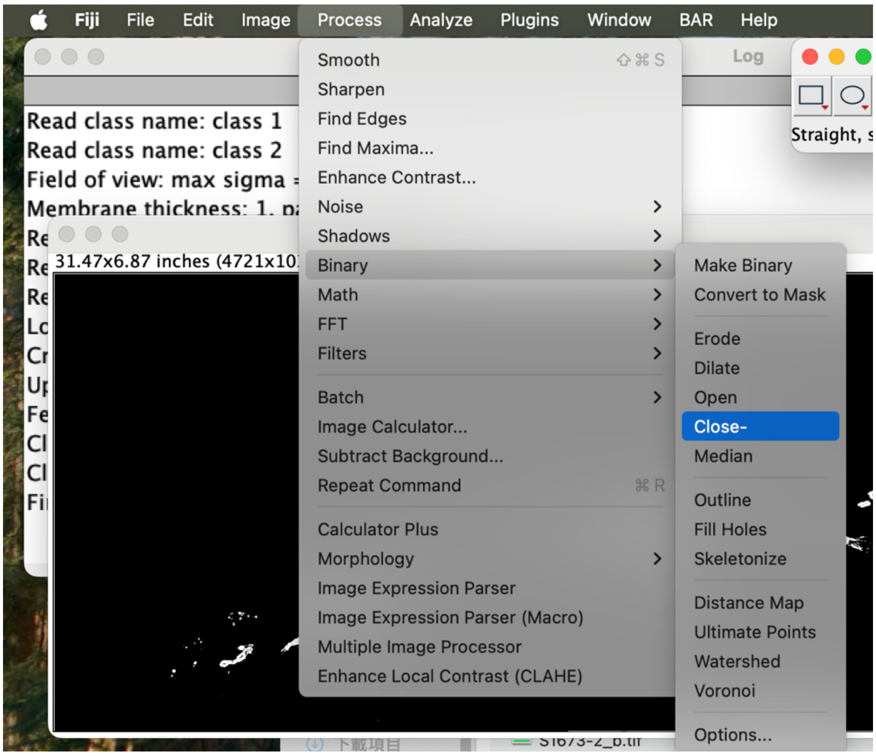

“Fill Holes”

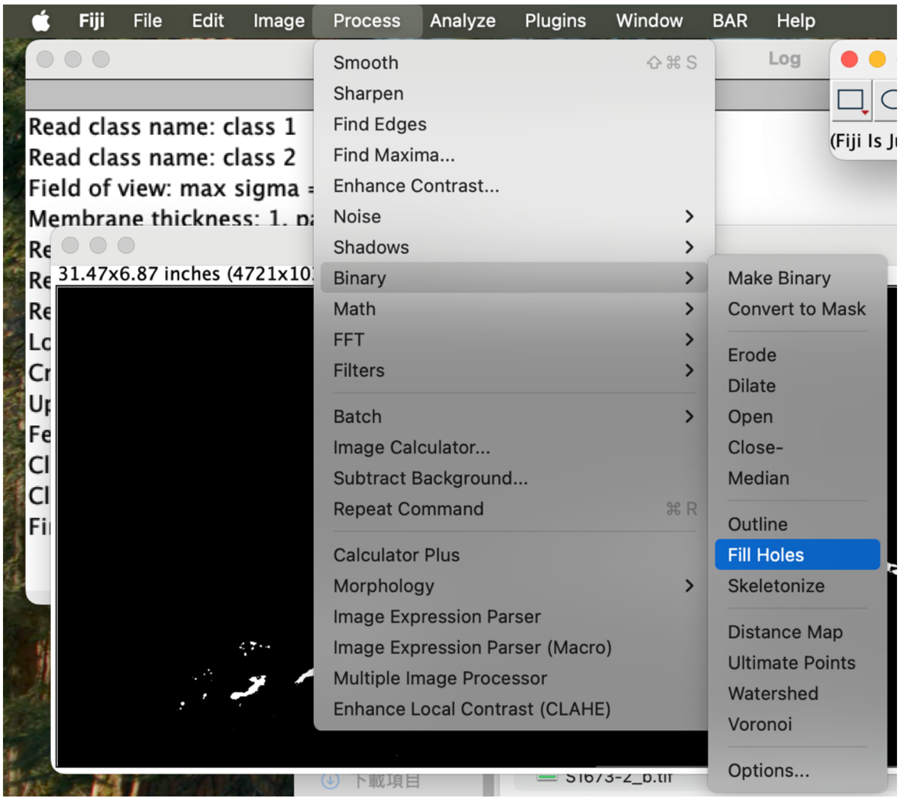

- Analyze the particles

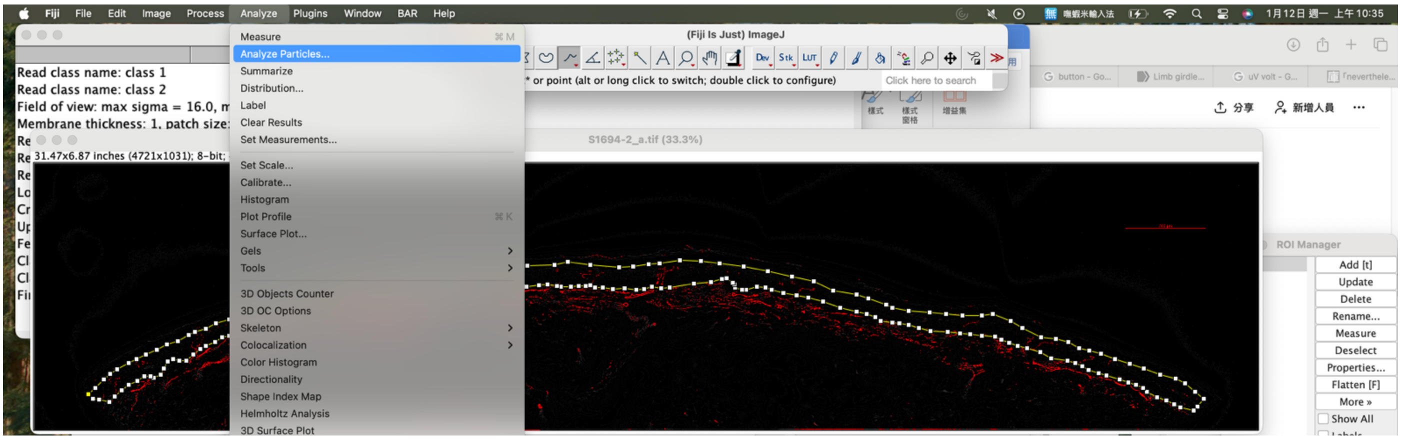

Set the details, and push “OK”

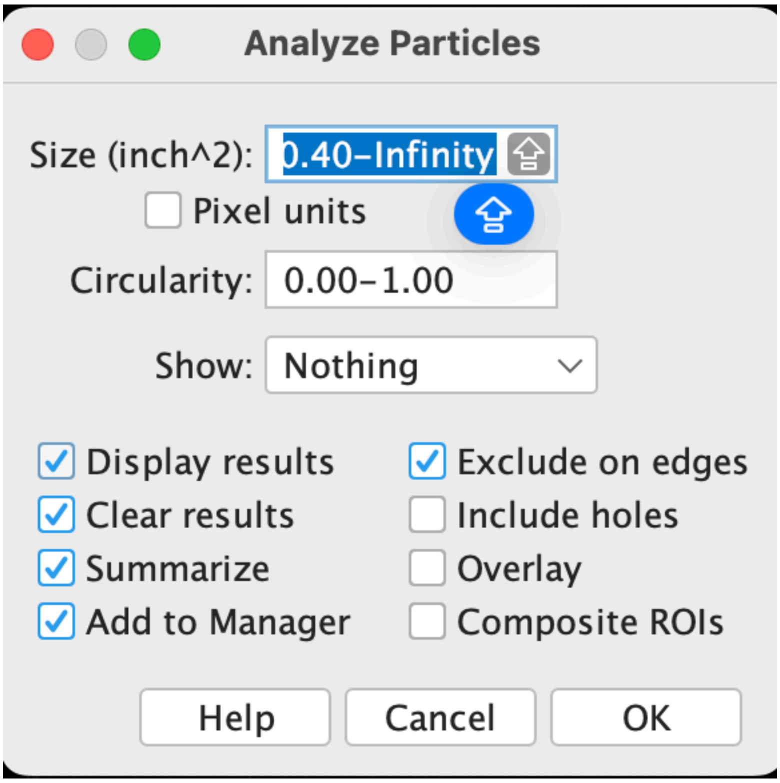

- Get the results (Total Area)

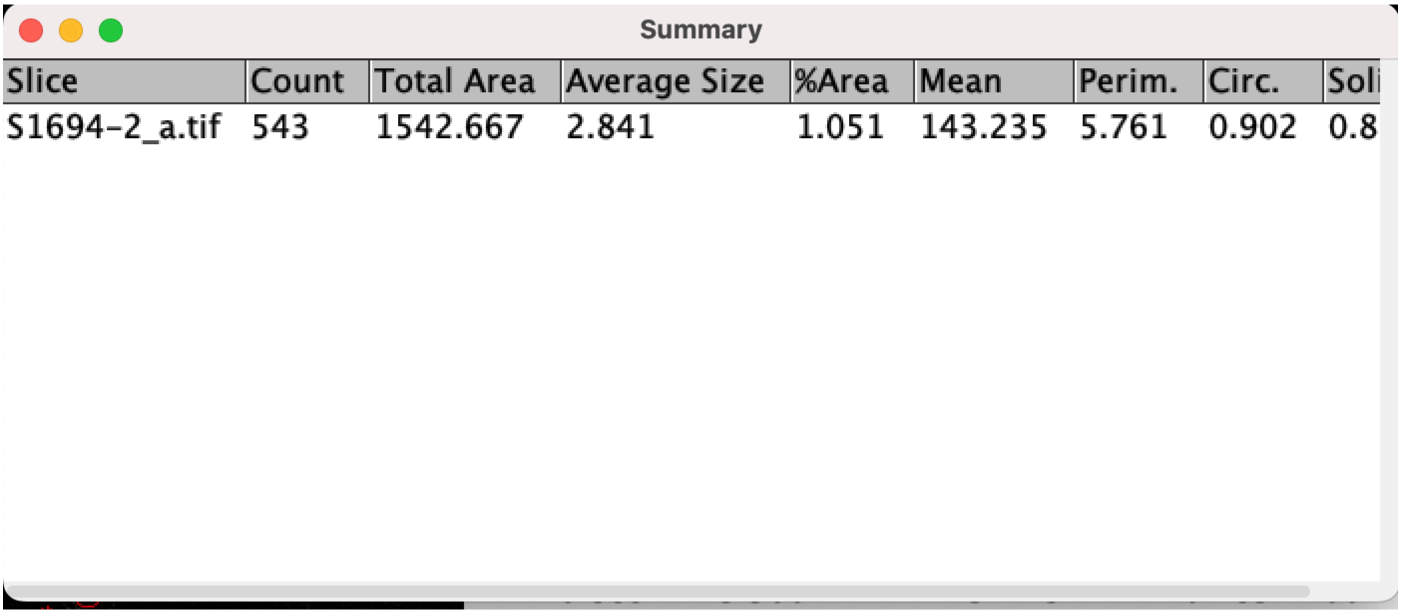

Summary

Slice	Count	Total Area	Average Size	%Area	Mean	Perim.	Circ.	Soli
S1694-2_a.tif	543	1542.667	2.841	1.051	143.235	5.761	0.902	0.8

ImageJ Macros used for IENF segmentation.

1. Custom-convolution filtering

```
run("Set Scale...", "distance=1.5 known=1 unit=micron global");

run("Convolve...", "text1=[0 0 -1 0 0\n0 -1 -1 -1    0\n-1 -1 12 -1 -1\n0 -1 -1 -1 0\n0 0 -1 0 0 \n] normalize");

run("Threshold...");

run("Manual Threshold", "min=61 max=255");

//You should adjust the threshold for selecting the IENF so that it matches your expectations. If the images are satisfactory, you can then run the macros below in one step to obtain the IENF area results.

setOption("BlackBackground", true);

run("Convert to Mask");

run("Close-");

run("Fill Holes")

roiManager("Select", 0);

run("Analyze Particles...", "size=0.40-Infinity display exclude clear summarize")

roiManager("Delete");

run("Close All");
```

2. Edge detection:

```
run("Set Scale...", "distance=1.5 known=1 unit=micron global");

run("Find Edges");

run("Manual Threshold", "min=50 max=255");

//You should adjust the threshold for selecting the IENF so that it matches your expectations. If the images are satisfactory, you can then run the macros below in one step to obtain the IENF area results.

setOption("BlackBackground", true);

run("Convert to Mask");

run("Close-");

run("Fill Holes")
```

```
roiManager("Select", 0);

run("Analyze Particles...", "size=0.40-Infinity display exclude clear summarize")

roiManager("Delete");

run("Close All");
```

3. Weka-based automated annotation:

```
run("Set Scale...", "distance=1.5 known=1 unit=micron global");

run("Trainable Weka Segmentation");

//It is recommended to divide the process into two steps, since ImageJ may not be able to execute both steps simultaneously in a single process.

call("trainableSegmentation.Weka_Segmentation.loadClassifier", "/Users/hsueh-wenhsueh/Desktop/Weka3_6.model");

// Press the “Create result” bottom to get the first segmented image.

// After the first segmentation, you can use the “Toggle Overlay” function to check whether the segmented images match your expectations. If not, you may use the selection tools to add the biased regions to either “Add to class 1” or “Add to class 2” accordingly, and then press “Train Classifier” to generate a new segmented image. This process can be repeated until the segmented images are satisfactory. If the images are satisfactory, you can then run the macros below in one step to obtain the IENF area results.

selectImage("Trainable Weka Segmentation v4.0.0");

close;

selectImage("Classified image");

run("8-bit");

run("Manual Threshold...", "min=61 max=128");

roiManager("Select", 0);

run("Analyze Particles...", "size=0.40-Infinity display exclude clear summarize")

setOption("BlackBackground", true);

run("Convert to Mask");

run("Invert");
```

roiManager("Delete");

4. Measurement of the area and perimeter of epidermis

//Drag the epidermis ROI into the ImageJ directly, not in the roiManager tool. You can then run the macros below in one step to obtain the the area and perimeter of epidermis.

```
run("Set Scale...", "distance=1.5 known=1 unit=micron global");

roiManager("Add");

roiManager("Select", 0);

run("Set Measurements...", "area mean min perimeter shape area_fraction redirect=None decimal=3");

roiManager("Measure");

roiManager("Delete");

run("Close All");
```
